# Advances in Catalysis Using N-Heterocyclic Carbene Platinum Complexes

**DOI:** 10.3390/molecules31030448

**Published:** 2026-01-27

**Authors:** Anna Smoczyńska, Sylwia Ostrowska, Cezary Pietraszuk

**Affiliations:** Faculty of Chemistry, Adam Mickiewicz University, Uniwersytetu Poznańskiego 8, 61-614 Poznań, Poland; anna.smoczynska@amu.edu.pl (A.S.); sylwia.ostrowska@amu.edu.pl (S.O.)

**Keywords:** platinum, N-heterocyclic carbene complexes, homogeneous catalysis

## Abstract

Apart from in hydrosilylation, platinum has traditionally played a limited role in homogeneous catalysis due to its high thermodynamic stability and lower intrinsic reactivity compared to other group 10 metals. However, the emergence of N-heterocyclic carbene (NHC) ligands has substantially broadened the catalytic profile of transition metals by enabling access to new mechanistic pathways and enhancing robustness under demanding conditions. This review summarizes advances in Pt–NHC catalysis reported between 2010 and 2025. These transformations encompass hydrosilylation of amides and CO_2_, hydroboration and diboration, hydroamination, alkyne hydration, hydrogenation, selective alkyne dimerization, Suzuki–Miyaura coupling, arene C–H borylation, and cycloisomerization reactions, in which NHC ligands enhance bond activation, control regio- and stereoselectivity, and stabilize reactive Pt intermediates, including chiral architectures, enabling high enantioselectivity.

## 1. Introduction

Research into the catalytic activity of platinum complexes remains significantly less common than research into other elements group 10 of the periodic table. This is primarily due to the higher thermodynamic stability and consequently lower intrinsic reactivity of platinum complexes, not to mention its high cost and scarcity. The current surge of interest in platinum N-heterocyclic carbene (NHC) complexes is largely driven by the search for complexes displaying improved anticancer activity [1,2,3]. Another important factor contributing to the growing interest in these compounds is their photophysical properties [4,5,6].

This review aims to present the progress in research on the catalytic activity of NHC platinum complexes that has been achieved between 2010 and 2025. A comprehensive description of platinum complex catalysis was presented by Clarke in 2001 [7]. The literature discussing the catalytic activity of platinum N-heterocyclic carbene (NHC) complexes up to 2009 has already been thoroughly summarized by Nolan [8]. Furthermore, Y. Fort and C. Comoy discussed this subject briefly in their book chapter [9]. More specialized reviews are referenced in the relevant sections of this manuscript.

Due to the abundance of review literature dedicated to the activity of Pt–NHC complexes in the hydrosilylation of carbon–carbon multiple bonds, e.g., [9,10,11,12,13], and the desire to focus attention on its other interesting forms of reactivity, hydrosilylation apart from the two more specific processes has not been discussed in this review.

## 2. Hydrosilylation of Amides

**Figure 1:**
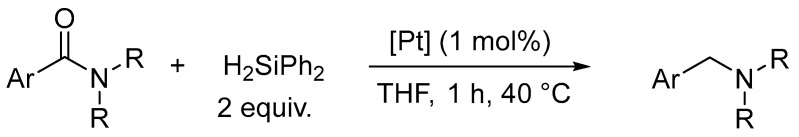
Reduction of tertiary amides to amines using H_2_SiPh_2_.

**Figure 2:**
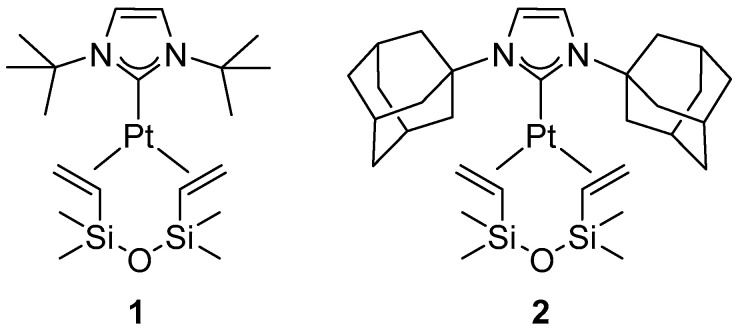
[Pt(ItBu)(dvtms)] (**1**) and [Pt(IAd)(dvtms)] (**2**)—active Pt–NHC catalysts for the reduction of tertiary amides to amines using H_2_SiPh_2_.

The hydrosilylation of amides represents a well-recognized method for the reduction of secondary and tertiary amides. In the literature, numerous examples employing complexes of various transition metals have been reported, including Ru [14], Zn [15,16], Mn [17], Ir [18], Ni [19], and Cu [20]. The reaction has also been the subject of a review article [21]. The first example of the catalytic activity of platinum compounds in this transformation was described in 2001 by Fuchikami [22], who demonstrated the activity of PtCl_2_ in the reduction of N-acetylpiperidine using triethylsilane. Subsequently, Nagashima demonstrated that commercially available platinum catalysts, such as the Karstedt catalyst and hexachloroplatinic acid, are catalytically active in the hydrosilylation of secondary amides to form the corresponding amines using disiloxanes, e.g., 1,1,3,3-tetramethyldisiloxane (TMDS) or 1,2-bis(dimethylsilyl)benzene [23]. In 2014, Beller and co-workers reported the first use of NHC platinum complexes in the reduction of secondary and tertiary amides to amines using diphenylsilane as a reducing agent [24]. The reduction of tertiary amides proceeded efficiently even at 40 °C, providing high conversions and selectivities (Figure 1). Among the platinum complexes tested, [Pt(ItBu)(dvtms)] (**1**) and [Pt(IAd)(dvtms)] (**2**) (Figure 2) gave the best results. Of all the silane reagents that were tested, diphenylsilane was found to be the most successful.

**Figure 3:**
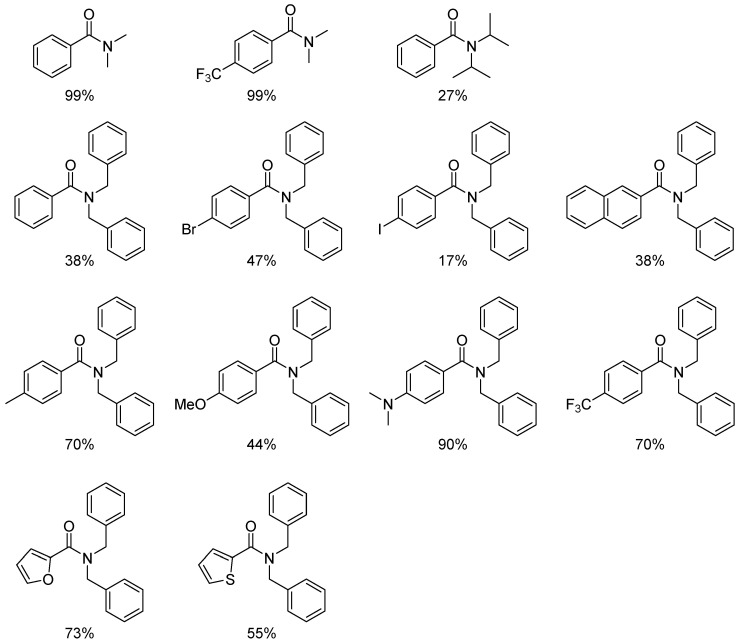
Reduction of amides with Ph_2_SiH_2_ catalyzed by [Pt(ItBu)(dvtms)]. The scope of the reaction. Reaction conditions: amide (0.5 mmol), [Pt] (1 mol%), Ph_2_SiH_2_ (2 equiv.), THF (3 mL), 40 °C, 1 h.

The reaction yield depends on the substituents on the nitrogen atom. Dimethylamides gave higher yields than dibenzylamides and the least active diisopropylamides. In the case of dibenzylamides, the presence of substituents R = Me, CF_3_ or NMe_2_ in the para position resulted in the formation of the desired amines with high yields. In the absence of a substituent in the para position (R = H) or in the presence of halides (R = I, Br), a significantly lower yields were observed. As shown in Figure 3, the heteroaromatic amides studied underwent smooth reduction. Among the investigated silanes, H_2_SiPh_2_ proved the most effective; mono- or trialkylsilanes resulted in significantly lower yields.

**Figure 4:**
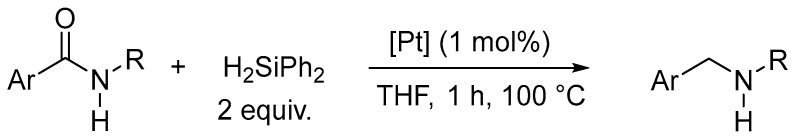
Reduction of secondary amides catalyzed by [Pt(ItBu)(dvtms)]. Reaction conditions: amide (0.5 mmol), [Pt] (1 mol%), Ph_2_SiH_2_ (2 equiv.), THF (3 mL), 100 °C, 1 h.

**Figure 5:**
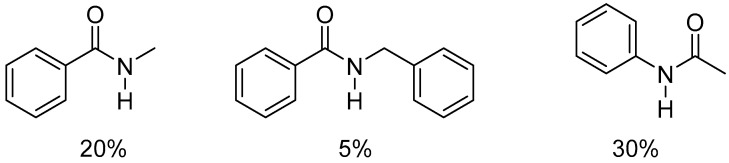
Secondary amides tested in reduction reactions with Ph_2_SiH_2_ and the yields obtained.

For secondary amides, the process required an elevated temperature of 100 °C and poor yields of between 5% and 30% were noted (see Figure 4 and Figure 5).

The authors suggest that the hydrosilylation of amides catalyzed by NHC platinum complexes occurs via a mechanism analogous to that observed with iridium and ruthenium complexes.

Recently, Nolan and Casin demonstrated that [Pt(DMS)_2_Cl_2_] (**3**) (where DMS = dimethyl sulphide) exhibits significantly higher catalytic activity in the reduction of N,N-dimethylacetamide with 1,1,3,3-tetramethyldisiloxane (TMDS) to form dimethylethylamine, compared to N-heterocyclic platinum complexes such as [Pt(IMes)(DMS)Cl_2_] (**4**), [Pt(ICy)(DMS)Cl_2_] (**5**) and [Pt(SIPr)(DMS)Cl_2_] (**6**). This was achieved using the Sunthetics ML platform [25]. [Pt(DMS)_2_Cl_2_] (**3**) used at a loading of 0.13 mol% enabled 92% conversion of the substrate under mild conditions (RT, 1 h), without the use of a solvent, achieving a TOF of 708 h^−1^. The study of the activity of complex **3** was unfortunately not conducted in the presence of amides that contain functional groups.

## 3. Hydrosilylation of CO_2_

**Figure 6:**
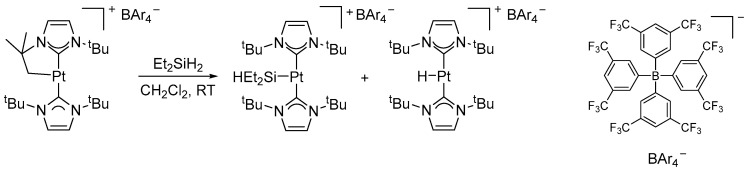
Reaction of the cationic platinum complex **7** with diethylsilane to form **8** and **9**.

**Figure 7:**
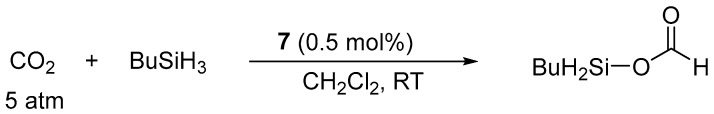
The formation of silyl formate through CO_2_ hydrosilylation using complex **7**.

Catalytic hydrosilylation of carbon dioxide is an attractive approach for the selective reduction of CO_2_ to formate, formaldehyde, methanol, or methane. Numerous transition metal systems have been reported, although control over product selectivity remains a major challenge. Various catalytic systems active in CO_2_ hydrosilylation were summarized in a review [26]. Rodríguez and Conejero demonstrated that the cationic complex [Pt(ItBu′)(ItBu)][BArF_4_] (**7**) (Figure 6) reacts with diethylsilane (H_2_SiEt_2_) to form stable and catalytically active species, namely, [Pt(SiEt_2_H)(ItBu)_2_][BArF_4_] (**8**) and [Pt(H)(ItBu)_2_][BArF_4_] (**9**). All three complexes catalyze the hydrosilylation of CO_2_, leading selectively to the corresponding silyl formates at room temperature (Figure 7) [27].

Complex **7** proved to be the most efficient of the three, whereas **8** and **9** exhibited comparable catalytic activity. When catalyst **7** (0.5%) was reacted with BuSiH_3_ under 5 atm of CO_2_, a clean reaction took place, leading almost exclusively to silylformate BuSiH_2_(OCOH) in around 15 min at room temperature. To avoid hydrolysis of the silanes, the reaction must be carried out under strictly anhydrous conditions. No side products such as methane or alcohols were detected. The calculated TON and TOF values were 200 and 714 h^−1^, respectively. By way of comparison, the most productive transition metal-based catalytic systems for the hydrosilylation of CO_2_ to form silylformates can achieve turnover numbers (TONs) of up to 70,000 [28].

## 4. Hydroboration

The hydroboration of unsaturated organic compounds represents a straightforward and efficient strategy for the synthesis of organoboron intermediates, which serve as versatile building blocks in organic and materials chemistry. These transformations are most frequently catalytic in nature, although uncatalyzed pathways have also been described. The current state of the art, including mechanistic aspects, substrate scope, and recent advances in transition metal catalysis, has been comprehensively summarized in recent reviews [29,30].

**Figure 8:**
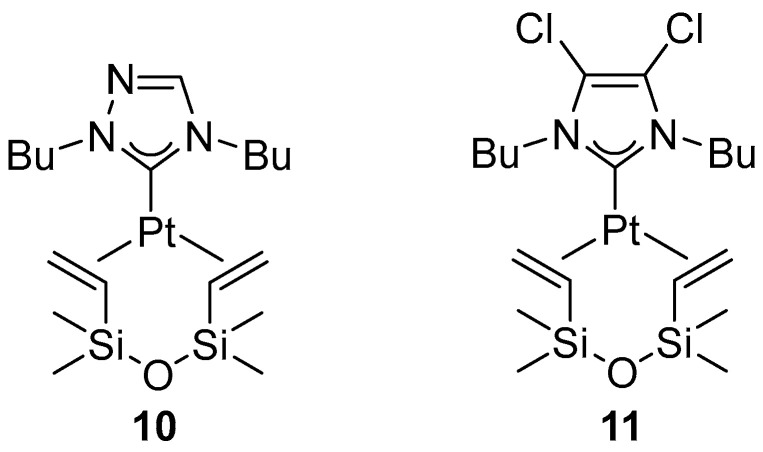
Pt–NHC complexes used by Fernandez and Peris in hydroboration of alkenes and alkynes.

In 2007, Fernandez, Peris, and co-workers demonstrated that stable platinum(0) bearing N-heterocyclic carbene (NHC) ligands are capable of promoting regioselective hydroboration of both alkenes and alkynes, exhibiting high activity and excellent control over product formation [31]. Among the tested NHC complexes, complexes **10** and **11** (Figure 8) demonstrated high catalytic activity combined with excellent chemo- and regioselectivity.

**Figure 9:**
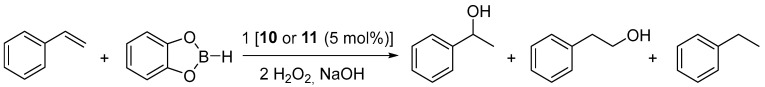
Platinum-catalyzed hydroboration of styrene with catecholborane and oxidation sequence.

The authors demonstrated that Pt–NHC complexes significantly outperform their phosphine-based analogues in terms of conversion, catalytic stability, and selectivity, achieving 85–90% selectivity for the α-addition product (Figure 9). Classical platinum precursors such as [Pt(PPh_3_)_4_] display only moderate catalytic activity, typically resulting in low conversions and poorly controlled product distributions, often accompanied by competitive alkene hydrogenation. These observations are attributed to the requirement for prior phosphine dissociation before entry into the productive catalytic cycle, as well as to the pronounced inhibitory effect of L-type phosphine ligands, which stabilize the platinum center at the expense of coordinative accessibility. Similarly, although Pt(COD)Cl_2_ is intrinsically more reactive, it fails to provide synthetically useful regioselectivity. Even small amounts of added phosphine lead to near-complete deactivation of the catalyst. Against this background, Pt–NHC complexes represent a qualitatively distinct class of catalysts. In contrast to phosphine-based systems, strongly bound NHC ligands maintain the Pt(0) center in a reactive state without the need for stepwise ligand dissociation, which translates into full conversions under mild conditions (room temperature, short reaction times). Notably, relative to the other platinum complexes examined in the study, only the Pt–NHC systems consistently exhibit a pronounced and reproducible preference for the branched product in the hydroboration of vinylarenes, pointing to more effective stabilization of benzylic intermediates in the key hydride or boryl migration step.

**Figure 10:**
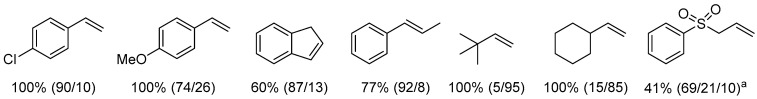
The scope of the platinum-catalyzed hydroboration of alkenes with catecholborane. The conversion values and the ratios of regioisomers (α-adduct/β-adduct) are presented. Reaction conditions: catalyst **10** (5 mol%), alkyne:borane = 1:1.1, THF, 25 °C, 3 h. ^(a)^ (α-adduct/β-adduct/hydrogenation product).

The substrate scope highlights clear structural limitations of the Pt–NHC system. Terminal vinylarenes are converted most efficiently, providing high conversions and good selectivity for the branched isomer, particularly for electron-poor substrates, consistent with stabilization of cationic or π-delocalized intermediates. For internal vinylarenes, regioselectivity is largely retained but at the expense of significantly reduced conversions, reflecting increased steric hindrance and diminished alkene coordination to the platinum center. In contrast, aliphatic substrates reveal a fundamental weakness of the catalyst, as terminal alkenes undergo regioselectivity reversal toward the linear product, indicating the absence of benzylic stabilization in the key migratory step (Figure 10).

**Figure 11:**
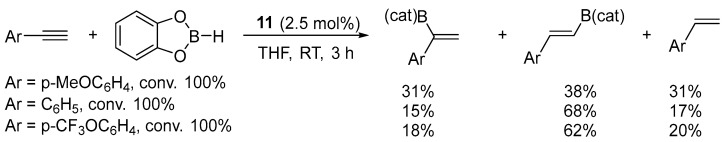
Hydroboration of terminal acetylene.

Complexes **10** and **11** remain active for several days and can be reused multiple times, without substantial loss of activity. Complexes **10** and **11** also exhibit catalytic activity in the hydroboration of alkynes (Figure 11). Hydroboration of alkynes, while rapid under mild conditions, affords mixtures of alkenylboronates with only moderate regio- and stereocontrol and no clear advantage over other platinum complexes, underscoring that vinylarenes, rather than alkynes, represent the most favorable substrate class for Pt–NHC catalysis.

Moreover, the authors demonstrated sequential hydroboration of an alkyne followed by in situ Suzuki–Miyaura coupling in a single reaction vessel, in the presence of complex **11**.

Compared to other NHC–M systems, particularly those based on copper, palladium, ruthenium, and rare earth metals, platinum Pt–NHC catalysts are less effective in terms of substrate scope and stereochemical control. Nevertheless, they facilitate high conversion and isolated yields, typically ranging from 70 to 95%, in the hydroboration of vinylarenes [31]. Furthermore, Pt–NHC complexes exhibit greater activity and selectivity than simple phosphine complexes [29,30].

## 5. Diboration

The catalytic addition of diboranes to internal and terminal alkynes in the presence of a platinum catalyst, affording cis-diboration products, was first reported by Miyaura in 1993 [32].

Over the following years, Pt(0) complexes were confirmed to be the most effective catalysts for diboration reactions. The literature describes numerous transition metal complexes that demonstrate catalytic activity in the diboration of alkenes, alkynes, and dienes. Comprehensive discussions of the reaction can be found in several review articles [33,34,35,36].

**Figure 12:**
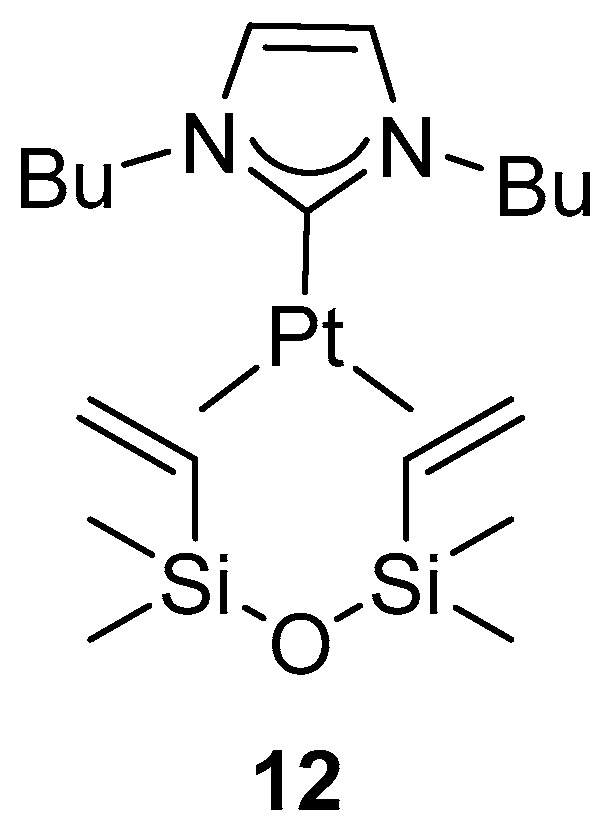
Pt–NHC complex active in diboration of alkynes and alkenes.

Mata and Fernández demonstrated that N-heterocyclic carbene (NHC) platinum complexes **10** and **11** (Figure 9), as well as **12** (Figure 12), exhibit catalytic activity in the diboration of both alkynes and alkenes [37].

Solvent-free diboration of both internal and terminal alkynes with B_2_cat_2_ in the presence of catalyst **10** resulted in conversions to cis-alkenyl bis(boronate) esters exceeding 95%. Using B_2_pin_2_ as a boronating reagent leads to a significant decrease in conversion: 3% for phenylacetylene and 60% for PhCCPh. In the diboration of terminal olefins, conversions ranging from 85% to 99% were achieved, but with significantly lower selectivity. The 1,2-diboration of aryl allylic sulfones with a twofold molar excess of B_2_pin_2_ yielded complete conversion and 90–94% selectivity. In this reaction, catalysts **10**–**12** exhibited comparable activity.

**Figure 13:**
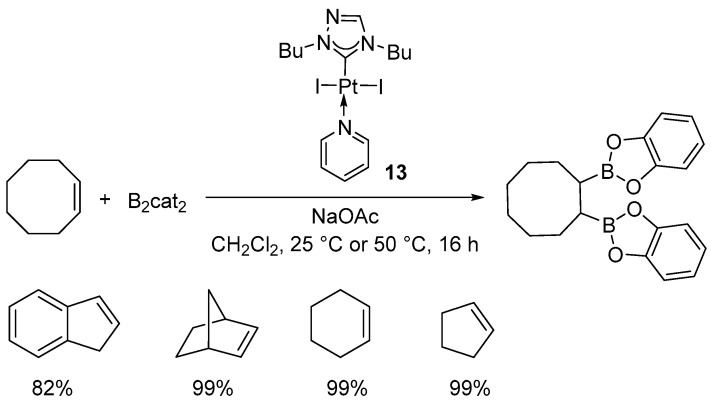
Catalytic diboration of cycloolefins with B_2_(cat)_2_ and the scope of the reaction. Reaction conditions: [Pt] (2 mol %), B_2_cat_2_ (2.1 equiv.), base (1 equiv.), CH_2_Cl_2_, 25 °C or 50 °C, 16 h.

Complex [Pt(NHC)(py)I_2_], was reported to be an active catalyst for the diboration of cycloolefins, allowing nearly complete conversion of cyclooctene, cyclopentene, norbornene, and cyclohexene, even at 25 °C (Figure 13) [38]. The optimal base required for catalysis was found to be NaOAc.

**Figure 14:**
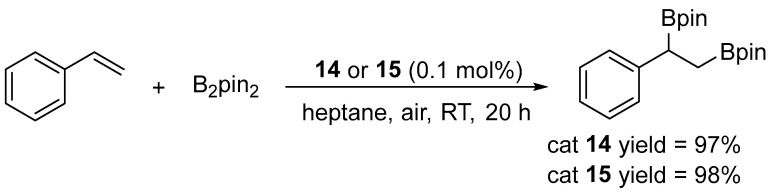
Diboration of styrene catalyzed by Pt–NHC complexes. Reaction conditions: [Pt] (1 mol–0.025 mol%), styrene (1 equiv.), B_2_pin_2_ (1.1 equiv.), heptane, 25 °C, 20 h.

Asachenko and co-workers synthesized a library of Marko-type platinum complexes, [Pt(NHC)(dvtms)], which were subsequently evaluated in the diboration of styrene (Figure 14) [39].

**Figure 15:**
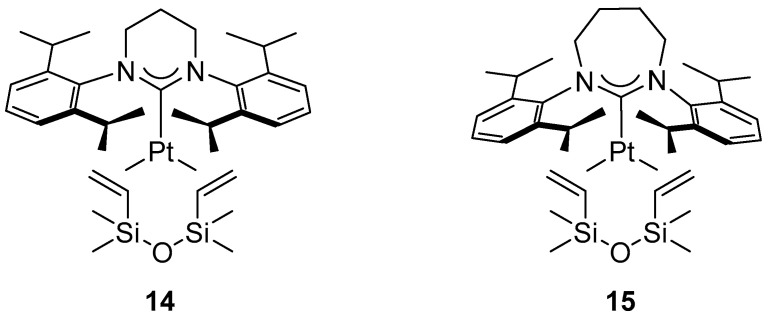
Platinum NHC complexes exhibiting the highest activity in the diboration of styrene.

The reaction proved to be highly sensitive to the steric properties of the catalyst. Depending on the complex employed, the yields varied from 0% to 99%. Expansion of the NHC ring from a five-membered to a six- or seven-membered framework caused the aryl substituents of the ligand to shift toward the metal center, resulting in increased steric congestion within the coordination sphere and, consequently, an enhancement in catalytic activity. The complex [Pt(7-Dipp)(dvtms)] (**15**) (Figure 15) exhibited exceptional performance, achieving nearly complete conversion (95%) even at a catalyst loading as low as 0.0025 mol %, which corresponds to a TON value of 3800.

**Figure 16:**
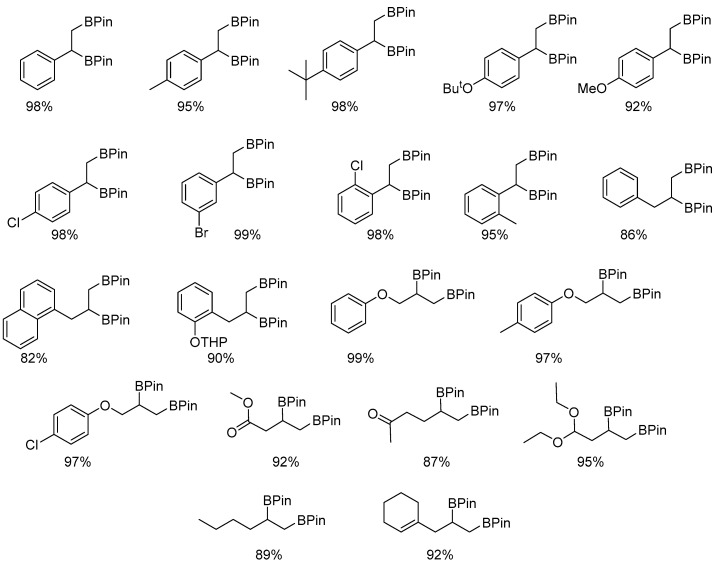
The scope of organoboron products of alkene diboration in the presence of **15**. Reaction conditions: **15** (0.5 mol%), alkene (1 equiv.), B_2_pin_2_ (1.1 equiv.), RT, 20 h.

In the presence of complex **15**, the scope of the reaction was examined, affording yields in the range of 82% to 99% (Figure 16). The catalysts displayed excellent functional group tolerance, accommodating both electron-donating and electron-withdrawing substituents. Aliphatic alkenes provided yields of 89–92%, whereas compounds bearing ester, ketone, or acetal functionalities afforded products at an 87–95% yield. All catalytic reactions were conducted in heptane, under air, at room temperature [39].

## 6. Hydroamination

Hydroamination is the addition of an N–H bond across an unsaturated hydrocarbon. This transformation is characterized by a high activation barrier, making it difficult to achieve in the absence of a catalyst. Numerous examples of transition metal complexes active in this reaction have been reported in the literature, and the details are comprehensively discussed in several review articles [40,41,42,43,44,45].

**Figure 17:**
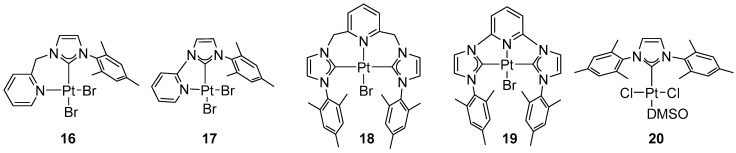
Complexes active in the hydroamination of ethylene reported by Limbach.

In 2012, Limbach and co-workers developed a series of platinum–NHC complexes (Figure 17) [46].

**Figure 18:**
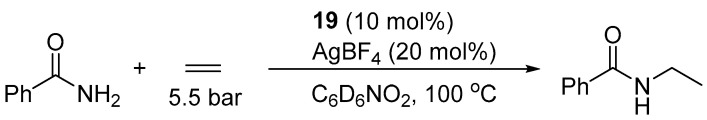
Ethylene hydroamination catalyzed by Pt–NHC complexes.

The synthesized complexes displayed excellent catalytic performance in ethylene hydroamination (Figure 18).

**Figure 19:**
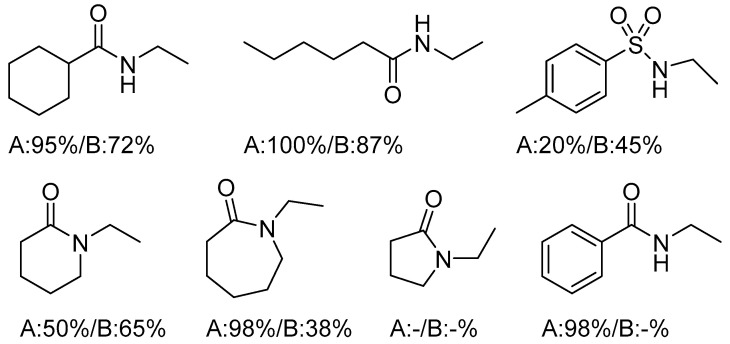
The scope of the hydroamination of ethylene by platinum complexes. Reaction conditions A: **19** (10 mol %), AgBF_4_ (20 mol %), nitrobenzene-d5 (0.5 mL); B: **17** (10 mol %), AgBF_4_ (10 mol %) and nitrobenzene-d5 (0.5 mL); GC conversions are given.

The reactions were conducted in nitrobenzene in the presence of silver salts at 150 °C. These conditions afforded the highest conversions (92–100%) and excellent selectivity. For reactions catalyzed by complex **17**, a beneficial effect of water on the hydroamination yield was observed when 0.5–1.0 equiv. relative to the catalyst was added; however, adding an excess of water (≥2 equiv.) led to catalyst deactivation. The highest yields were obtained for amides, while sulfonamides exhibited the greatest selectivity. Alkylamides reacted readily, and the highly reactive substrates cyclohexanecarboxamide and heptamide were hydroaminated twice to give *N*,*N*-diethylamides. For cyclic amines, the reactivity was governed primarily by the ring size and the nature of the catalyst. In contrast, strong nucleophiles such as ammonia, aniline, or morpholine did not undergo hydroamination under these conditions. The hydroamination of propylene and styrene proceeded with lower efficiency compared to ethylene (Figure 19) [46].

Within the investigated series, a pronounced effect of ligand architecture is observed: chelating and pincer-type complexes exhibit markedly higher catalytic activity and stability than their neutral or monodentate analogues, which remain catalytically inactive even upon attempted cationization. These differences indicate that the performance of Pt–NHC systems is governed not solely by the strong σ-donor character of the NHC ligand, but more critically by the ability of the ligand framework to stabilize a highly electrophilic platinum center while maintaining an accessible coordination site.

At the same time, the methodology is subject to clear limitations. Catalytic activity relies on the in situ generation of active species using silver salts and non-coordinating anions, which narrows the operational window and diminishes the practical utility of the system. Moreover, the substrate scope is restricted to weakly basic nucleophiles; more basic amines lead to catalyst deactivation through the formation of persistent Pt–C intermediates or competing side reactions.

**Figure 20:**
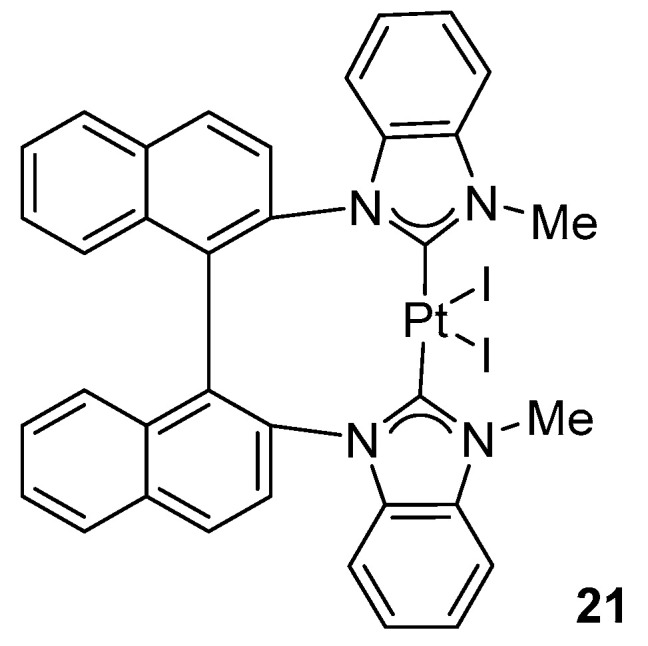
Pt-bis-carbene complex exhibiting activity in intramolecular hydroamination.

Xu and Shi synthesized a Pt(II) complex bearing a chelating bis-carbene ligand (Figure 20) [47].

Complex **21** was tested in intramolecular hydroamination of substrates bearing both an amine functionality and an unsaturated bond.

A key observation of this study is that pre-catalyst **21** is intrinsically inactive; efficient hydroamination is only achieved upon in situ generation of a cationic platinum species in the presence of AgBF_4_ (6 mol%), which already limits the practical applicability of the method at the design stage.

**Figure 21:**
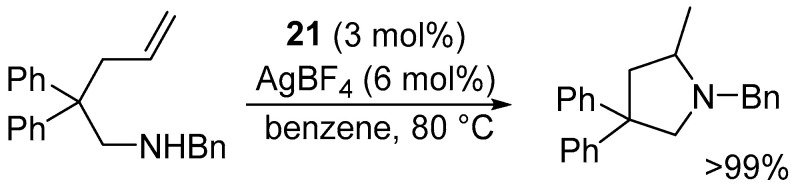
Intramolecular hydroamination catalyzed by **21**.

Under optimized conditions (3 mol% 3 and 6 mol% AgBF_4_ in benzene at 80 °C for 48 h), very high yields were obtained (up to 99%) (Figure 21). Nevertheless, the reaction profile reveals significant practical limitations, including the requirement for elevated temperatures (no conversion at 20 °C and only ca. 40% at 60 °C) and prolonged reaction times (ca. 45% yield after 24 h). Furthermore, the result is sensitive to reaction parameters such as the nature of the solvent and counterion [47].

**Figure 22:**
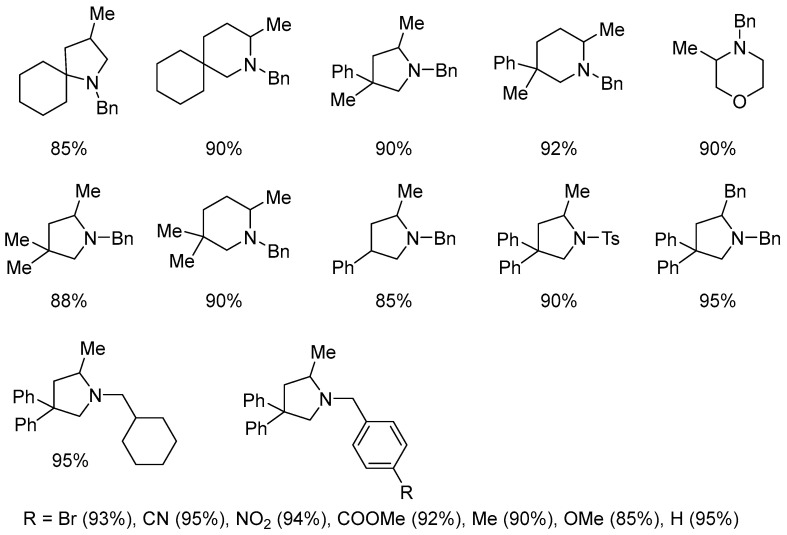
Intramolecular hydroamination of inactivated alkenes in the presence of pre-catalyst **21** along with AgBF_4_. The scope of the reaction.

The catalytic system tolerates a range of functional groups (such as Br, CN, NO_2_, esters, Me, OMe) (Figure 22). *N*-Boc-protected amides fail to undergo cyclization, whereas the more electron-withdrawing and less sterically demanding tosyl protecting group remains compatible.

**Figure 23:**
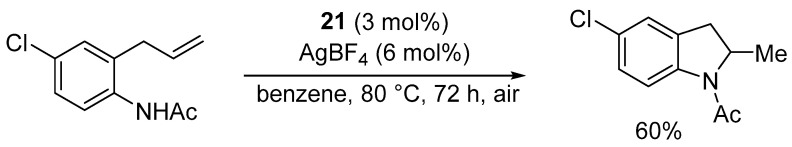
Intramolecular hydroamination of *N*-(2-allyl-4-chlorophenyl)acetamide catalyzed by **21**/AgBF_4_.

Furthermore, this complex, along with AgBF_4_, was active in the intramolecular hydroamination of *N*-(2-allyl-4-chlorophenyl)acetamide to give the corresponding indole at moderate yield in the presence of 3 mol % of pre-catalyst **21** (Figure 23).

**Figure 24:**
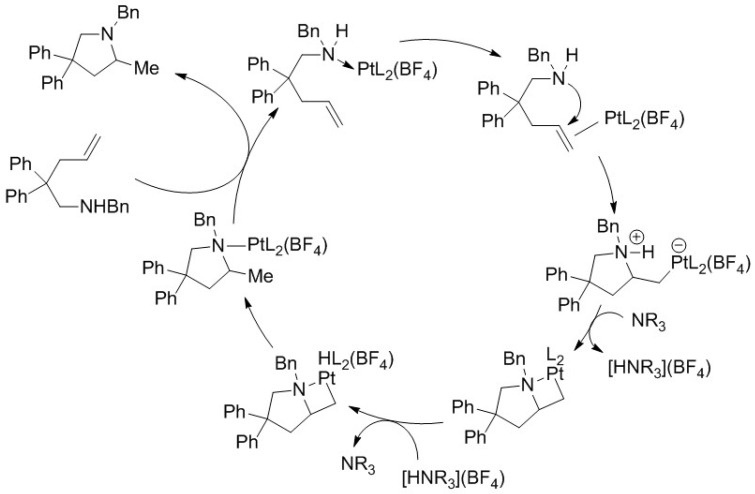
Plausible mechanism of intermolecular hydroamination [47].

The authors propose that the reaction proceeds via in situ generation of a cationic platinum species, followed by alkene coordination and nucleophilic attack of the amine to form a zwitterionic intermediate, which subsequently undergoes oxidative addition and reductive elimination to regenerate the active Pt(II) catalyst (Figure 24). This mechanistic scenario is inferred primarily from catalytic trends and the requirement for silver salts, while no direct experimental evidence is provided to unequivocally support the proposed pathway [47].

**Figure 25:**
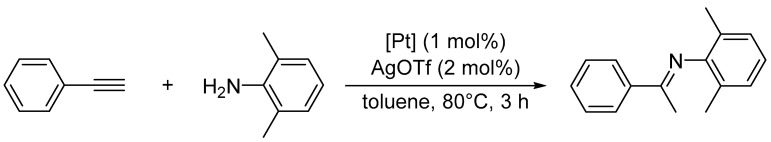
Hydroamination of phenylacetylene with 2,6-dimethylaniline. Reaction conditions: [Pt] (1.0 mol%), AgOTf (2 mol%), acetylene (2 equiv.), aniline (1 equiv.), 80 °C, 3 h, dry nitrogen.

**Figure 26:**
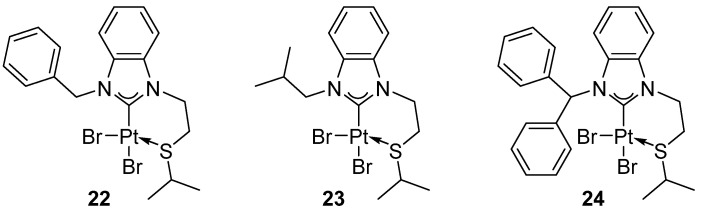
Benzimidazolin-2-ylidene complexes with thioether functions containing a six-membered chelate ring [48].

Huynh reported hydroamination of phenylacetylene, selectively affording the Markovnikov product (Figure 25), in the presence of platinum(II) complexes with thioether-functionalized benzimidazolin-2-ylidene ligands (Figure 26) [48].

Complexes in the absence of an activator remain inactive, whereas the addition of AgOTf (2 mol%) enables the formation of 31–87% (GC yield) of hydroamination products (Figure 25) depending on the catalyst used. In this series of catalysts, the ligand architecture has a clear impact on catalytic performance; the chelate forming a six-membered ring provides the highest activity (up to 87%) whereas complexes with a longer tether are less efficient (31–61%). Another factor determining the activity of the catalyst is the steric properties of the NHC ligand.

**Figure 27:**
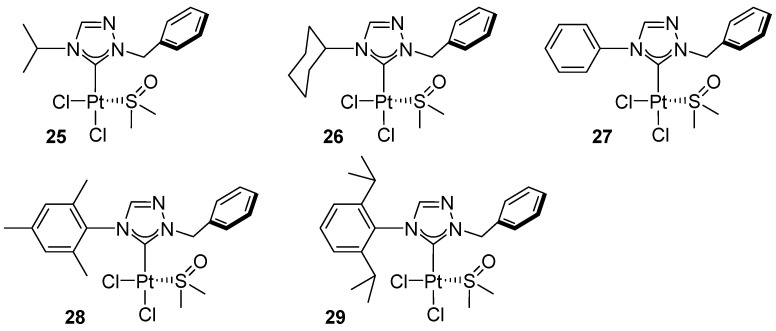
cis-[PtCl_2_(1,2,4-triazolin-5-ylidene)(DMSO)] complexes studied by Nguyen.

**Figure 28:**
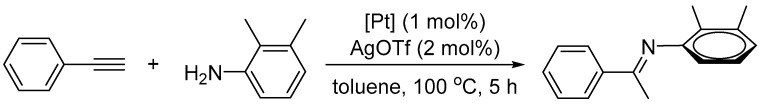
Hydroamination of phenylacetylene with 2,3-dimethylaniline.

Nguyen and co-workers demonstrated that Pt(II) complexes bearing 1,2,4-triazolin-5-ylidene ligands, [PtCl_2_(1,2,4-triazolin-5-ylidene)(DMSO)], differing in their N4 substituents (Figure 27), are catalytically active in the intermolecular hydroamination of phenylacetylene with aromatic amines in the presence of AgOTf, affording products at 27–59% yield (Figure 28) [49].

**Figure 29:**
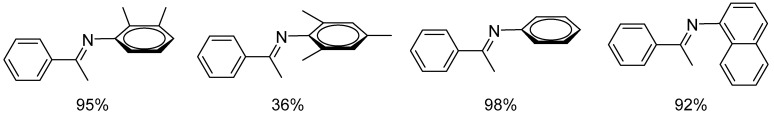
The scope of products of phenylacetylene hydroamination with various amines, catalyzed by cis-[PtCl_2_(1,2,4-triazolin-5-ylidene)(DMSO)].

In subsequent experiments, the hydroamination of phenylacetylene with various aromatic amines was carried out using the most active complex, cis-[PtCl_2_(1,2,4-triazolin-5-ylidene)(DMSO)], which afforded products at yields of up to 98% (Figure 29).

Within the catalyst series, a clear dependence on the nature of the NHC ligand is observed, with the authors ranking the activities as **29** < **28** < **25** < **27** < **26**. The most active complex, **26**, affords up to 95% yield of product under more forcing conditions (120 °C, 12 h). Studies of the substrate scope reveal pronounced steric sensitivity of the reaction, as evidenced by the markedly lower yield obtained with 2,4,6-trimethylaniline (36%). On the other hand, less sterically hindered amines, such as aniline and 1-naphthylamine, react efficiently, yielding 98% and 92% of the hydroamination product, respectively.

In summary, Pt–NHC complexes are competent catalysts for hydroamination, offering high regioselectivity and good functional group tolerance in selected systems. However, their practical application is limited by the need for catalyst activation, the requirement for elevated temperatures, and sensitivity to reaction parameters. Sterically demanding or weakly nucleophilic substrates are inactive in the presence of Pt–NHC, and asymmetric variants typically exhibit insignificant enantiomeric induction. By contrast, Ru-, Pd- and Rh-based catalysts generally demonstrate higher intrinsic activity, a broader substrate scope and more favorable operating conditions.

## 7. Hydration of Alkynes

The catalytic addition of water to alkynes is an atom-economical and environmentally sustainable method for C–O bond formation. A wide range of transition metal complexes, including Pt, Ag, Au, Ru, Os, Rh, and Ni, exhibit catalytic activity in this transformation. The latest knowledge on the hydration of alkynes catalyzed by NHC transition metal complexes has been summarized in recent reviews [50,51,52].

**Figure 30:**
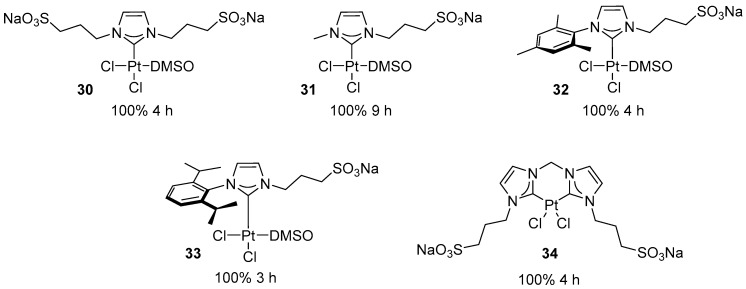
Water-soluble platinum NHC complexes synthesized by Flores and Jesús.

Platinum(II) complexes rank among the most efficient transition metal catalysts for the hydration of alkynes according to Markovnikov’s rule. The first platinum(II)-catalyzed alkyne hydration was reported by Chatt and Duncanson, who employed Na_2_PtCl_4_·H_2_O in ethanol solution [53]. Subsequently, Jennings and co-workers demonstrated that Zeise’s dimer and Pt(II) halides are effective catalysts for the addition of water to electron-rich terminal and internal alkynes [54,55,56]. Later, Atwood reported the hydration of alkynols using platinum(II) complexes bearing water-soluble sulfonated phosphine ligands, broadening the substrate scope of platinum-based hydration catalysis [57,58]. In 2013, Flores and Jesús reported for the first time the application of Pt–NHC complexes in the hydration of alkynes [59]. The authors synthesized a series of sulfonated, water-soluble NHC complexes (Figure 30).

**Figure 31:**
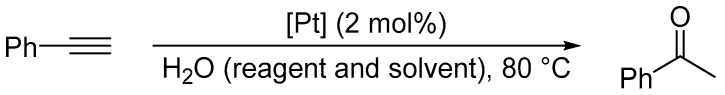
Hydration of phenylacetylene with water. Reaction conditions: [Pt] (2 mol%), H_2_O (used as a reagent and solvent), 80 °C.

Complexes **30**–**34** were shown to catalyze the hydration of phenylacetylene in water under acid-free conditions and with moderate metal loading (Figure 31). Complex **31**, which contains a small methyl substituent on the nitrogen atom of the NHC ligand, exhibits significantly lower activity than complexes **30**, **32,** and **33**.

**Figure 32:**
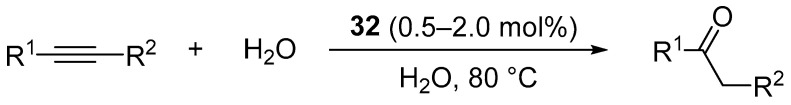
Hydration of acetylenes in the presence of soluble NHC platinum complexes. Reaction conditions: 80 °C, H_2_O used as solvent, [Pt] (2 mol%).

**Figure 33:**
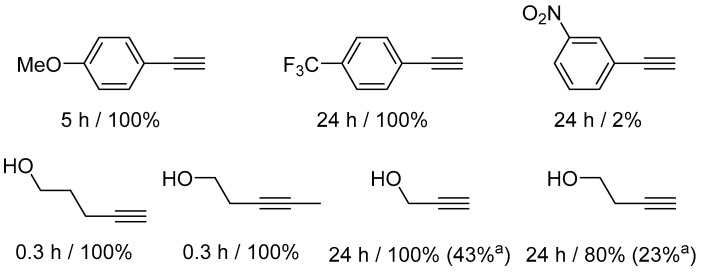
The scope of hydration of acetylenes in the presence of water-soluble NHC platinum complexes. Conversions determined by ^1^H NMR spectroscopy. ^(a)^ Ketone yield measured by ^1^H NMR spectroscopy using NEt_4_I as internal standard.

The hydration of a broader range of alkynes was examined in the presence of complex **32** (Figure 32 and Figure 33).

The results indicate a deactivating effect of electron-accepting groups in the hydration of arylacetylenes. 3-nitrophenylacetylene remains essentially unreactive. However, the poor solubility of some reagents does not allow reliable conclusions to be drawn. It has been observed, however, that alkynols are highly reactive in the presence of **32**. 3- and 4-pentynol undergo rapid hydration, which the authors explain by proposing the assistance of the OH group. However, shorter alkynols (propargyl alcohol, 3-butyn-1-ol) require a longer reaction time (24 h) and undergo competitive polymerization.

**Figure 34:**
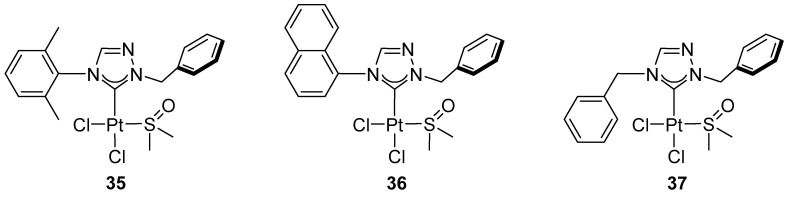
1,2,4-triazolin-5-ylidene platinum complexes described by Nguyen and Huynh.

In 2020, Nguyen and Huynh reported Pt(II) complexes bearing 1,2,4-triazolin-5-ylidene ligands **27**, **29** (Figure 27), and **35**–**37** (Figure 34), differing in their N4 substituents [60].

**Figure 35:**
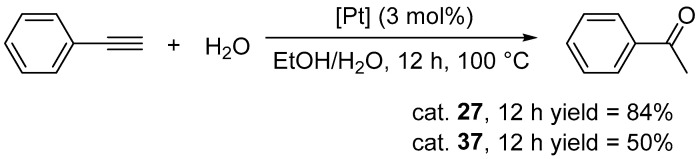
The hydration of phenylacetylene catalyzed by platinum triazolylidene complexes.

Complexes were tested in the hydration of phenylacetylene (Figure 35).

The complexes can catalyze the hydration of phenylacetylene, giving only Markovnikov’s product. Substantial variations in catalytic performance were observed as a function of ligand steric and electronic properties. Complexes **29** and **35** exhibited negligible activity (≤10% yield after 12 h at 100 °C), whereas complex **36** reached 19% under identical conditions. The highest activity was achieved with complex **27** (Figure 35).

Pt(II)–NHC complexes are catalysts whose efficiency is strongly dependent on the ligand architecture. Water-soluble, sulphonated Pt–NHC systems enable rapid, acid-free hydration in pure water, achieving approximately 90% conversion of phenylacetylene after 1 h and complete conversion within 3–4 h at 2 mol% Pt. This efficiency places them on par with the most effective classical Pt(II) catalysts, such as PtCl_2_, K_2_PtCl_4_/Na_2_PtCl_4_·xH_2_O, [PtCl_2_(C_2_H_4_)]_2_ (Zeise’s dimer) and [PtCl_2_(PR_3_)_2_], which typically provide 70–95% efficiency at 50–100 °C using 1–10 mol% Pt, often requiring organic cosolvents and/or extended reaction times (6–24 h) [54,55,56,57,58]. In contrast, triazolylidene-based Pt–NHC complexes exhibit significantly lower activity, requiring EtOH/H_2_O mixtures, elevated temperatures (80–100 °C), and extended reaction times to achieve yields up to 80–85% after 12–24 h at higher catalyst loadings.

Compared to catalysts based on other transition metals, Pt(II)-NHC complexes exhibit limited activity, yet demonstrate clear Markovnikov selectivity. Au(I)-NHC systems dominate in terms of rate and yield, enabling the acid-free hydration of alkynes at catalyst loadings in the ppm range. In contrast, Ag-NHC and Ru-NHC complexes demonstrate moderate performance and frequently necessitate additional activation. Cu–NHC, Pd–NHC, Ir, and Rh systems rarely favor classical alkyne hydration, instead favoring alternative hydrofunctionalization pathways. In this context, Pt–NHC catalysts are notable in that they enable hydration reactions to be carried out in an aqueous environment without the need for acid additives, while maintaining a high degree of selectivity.

## 8. Hydrogenation

**Figure 36:**
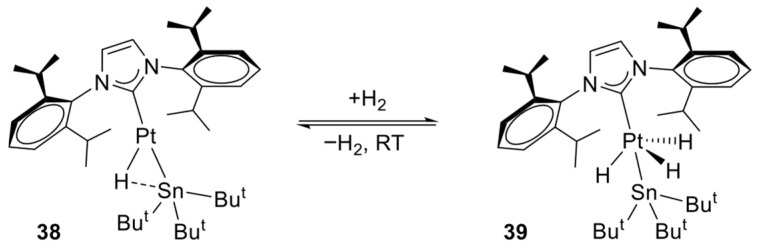
The oxidative addition of H_2_ to complex **38**.

Captain and co-workers reported that the reaction of [Pt(COD)_2_] with t-Bu_3_SnH and IPr affords the stable and catalytically active complex [Pt(IPr)(SnBu_3_)(H)] (**38**). This electronically unsaturated species undergoes reversible intramolecular C–H activation of the methyl groups of the NHC ligand. Furthermore, it reacts with H_2_ at room temperature to give a platinum(IV) trihydride complex, formed reversibly under mild conditions. The oxidative addition of H_2_ to complex **38** proceeds readily at room temperature (Figure 36).

The catalytic activity of the complex was evaluated in the hydrogenation of styrene to ethylbenzene, affording 33% conversion after 20 h under 1 atm of H_2_ at room temperature. Studies performed with styrene enabled the characterization of key intermediates, providing insight into the reaction mechanism. The addition of 1 equiv. of styrene led to the formation of [Pt(IPr)(SnBu_3_)(η^2^-CH_2_CHPh)(H)] (**40**), which likely corresponds to the first step of the catalytic cycle, whereas an excess of styrene yielded the inactive complex, [Pt(IPr)(η^2^-CH_2_CHPh)_2_] (**41**) [61].

No further catalytic studies have been conducted on this complex. The reaction conditions were not optimized and the TON/TOF values were not determined. Furthermore, the influence of key parameters such as substrate concentration, H_2_ pressure and substrate scope was not investigated. Therefore, the reported conversion should only be considered qualitative evidence of the validity of the alkene hydrogenation principle by a coordinatively unsaturated Pt–NHC moiety, and should not form the basis of direct comparisons with optimized hydrogenation catalysts.

## 9. Dimerization of Terminal Acetylenes

**Figure 37:**
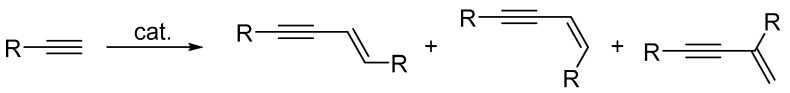
Dimerization of terminal acetylene (non-selective).

Dimerization of terminal alkynes (Figure 37) is a known method for the preparation of disubstituted 1,3-enynes. This catalytic reaction occurs effectively in the presence of many transition metal complexes, but depending on the catalyst used and the substituent at the carbon-carbon triple bond, different regio- and stereoselectivity is observed [62,63,64].

**Figure 38:**
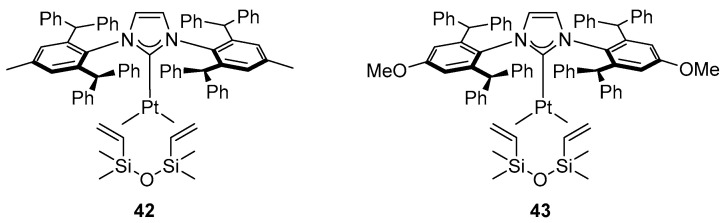
Platinum complexes with bulky NHC ligands.

**Figure 39:**
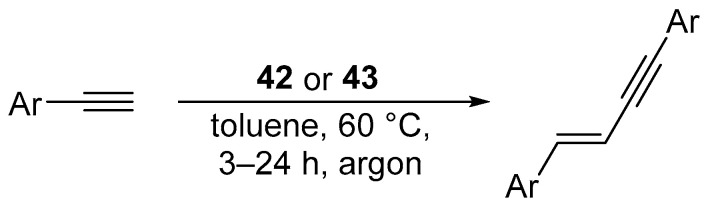
Platinum-catalyzed selective dimerization of terminal arylacetylenes using Markó-type NHC complexes **42** and **43**.

In 2017, Żak et al. reported activity of the Marko-type platinum complex [Pt(IPr*)(dvtms)] (**42**) or [Pt(IPr*^OMe^)(dvtms)] (**43**) (Figure 38) in the selective dimerization of terminal arylacetylenes (Figure 39) [65].

**Figure 40:**
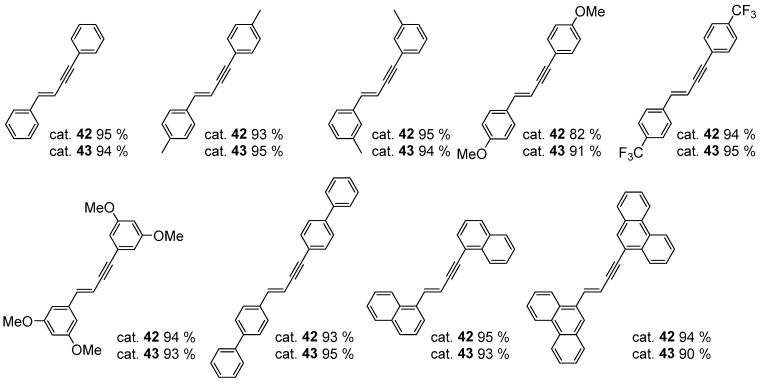
The scope of the platinum-catalyzed dimerization of terminal arylacetylenes.

The reaction was shown to proceed efficiently across a broad range of terminal arylacetylenes (Figure 40).

**Figure 41:**
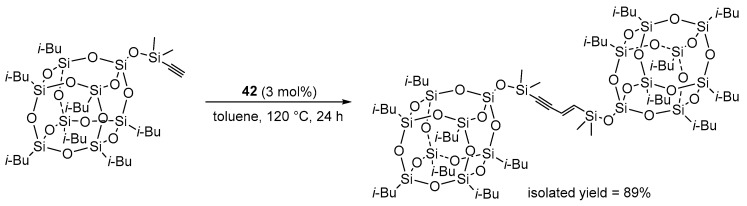
Exemplary dimerization of alkynylsiloxysilsesquioxane.

This reaction has also been successfully applied to the synthesis of silsesquioxane derivatives (Figure 41) [66].

These reactions are unique examples of catalytic dimerization of terminal acetylenes in the presence of platinum complexes. This demonstrates the ability of NHC ligands to facilitate new reaction pathways. Furthermore, most likely due to the steric properties of the NHC ligands, the reaction is completely regio- and stereoselective. It is notable that there are only a limited number of catalytic systems that enable the exclusive formation of a single isomer.

## 10. Suzuki–Miyaura Coupling

**Figure 42:**
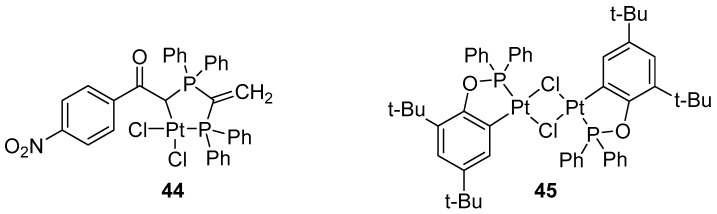
Platinum complexes **44** and **45** tested Suzuki–Miyaura coupling.

**Figure 43:**
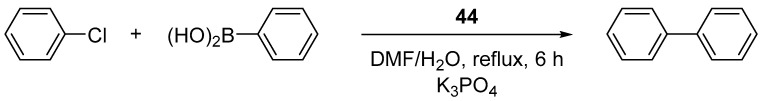
Synthesis of biphenyl via Suzuki–Miyaura coupling.

**Figure 44:**
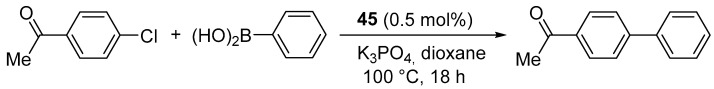
Suzuki–Miyaura coupling of 1-(4-chlorophenyl)ethan-1-one with phenylboronic acid.

The Suzuki–Miyaura cross-coupling reaction, in which boronic acids/esters react with organic halides, is typically catalyzed by palladium complexes. Efficient catalysis of the Suzuki–Miyaura cross-coupling has also been reported for other metals such as Ni, Cu, Co, Fe, Rh, and Ru [67]. The reaction has also been investigated in the presence of platinum complexes [31,68,69]. In 2007, Peris and Fernandes described coupling of alkenylboronic catechol esters with 4-methoxyphenyl iodide in the presence of complex **11** (Figure 9). Sabounchei reported the catalytic activity of complex **44** (Figure 42) in Heck and Suzuki–Miyaura couplings. In the coupling of chlorobenzene with phenylboronic acid, the use of complex **44** afforded the desired product in yields of up to 41% (Figure 43) [68]. Bedford demonstrated the high productivity of platinacyclic complexes (**45**, Figure 42) in the coupling of aryl bromides (TON up to 2,500,000), as well as their limited activity in the coupling of ClC_6_H_4_C(O)Me-4 (Figure 44) [69].

**Figure 45:**
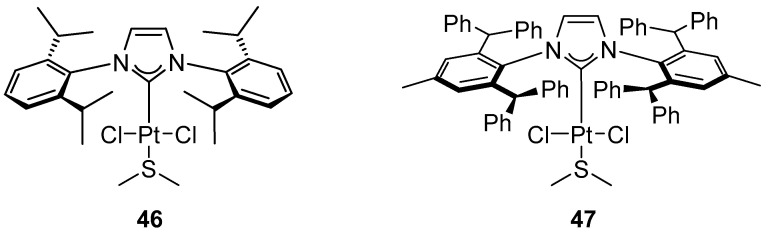
Platinum NHC complexes [Pt(IPr)(DMS)Cl_2_] (**46**) and [Pt(IPr*)(DMS)Cl_2_] (**47**) used in Suzuki–Miyaura reactions.

**Figure 46:**
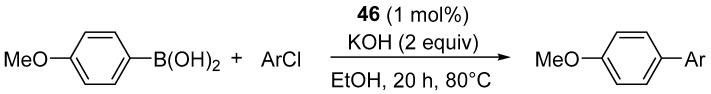
Suzuki–Miyaura coupling of aryl- and alkenylboronic acids with aryl chlorides catalyzed by complex **46**.

**Figure 47:**
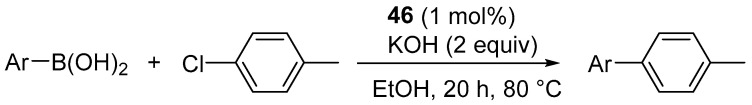
Additional examples of platinum-catalyzed Suzuki–Miyaura couplings, illustrating the influence of aryl chloride substitution patterns and boronic acid electronics on reaction efficiency.

Recently, Nolan and Pietraszuk evaluated the catalytic activity of the platinum complexes [Pt(IPr)(DMS)Cl_2_] (**46**) and [Pt(IPr*)(DMS)Cl_2_] (**47**), recently reported by Nolan (Figure 45) [70], in the coupling of aryl- and alkenylboronic acids with aryl chlorides (Figure 46 and Figure 47) [71].

Efficient reaction progress was observed when KOH was used as the base and EtOH as the solvent.

**Figure 48:**
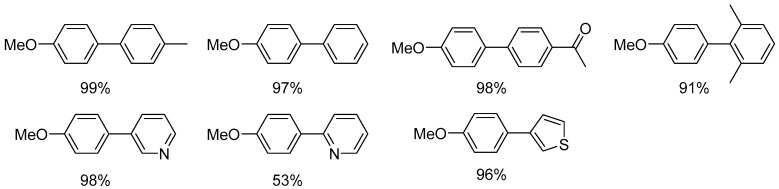
The scope of platinum-catalyzed Suzuki–Miyaura coupling of 4-methoxyphenylboronic acid with aryl chlorides.

**Figure 49:**
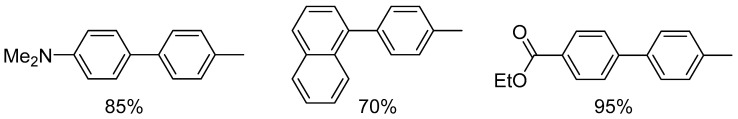
The scope of the platinum catalyzed Suzuki–Miyaura coupling of arylboronic acids with 4-chlorotoluene.

The reaction allowed for the formation of a wide scope of biaryls at high yields (Figure 48 and Figure 49).

**Figure 50:**
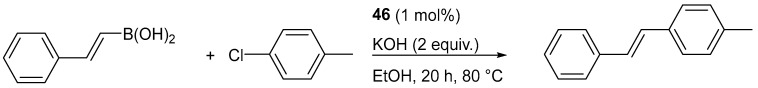
High-yielding coupling of vinylboronic acid with tolyl chloride catalyzed by platinum NHC complexes.

High yields were obtained for the coupling of vinylboronic acid with tolyl chloride (Figure 50).

**Figure 51:**
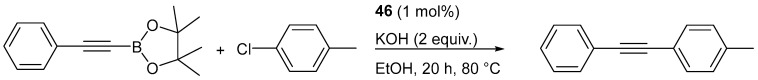
Suzuki–Miyaura coupling of phenylethynylboronic acid pinacol ester with aryl chlorides, showing low yields for ethynyl-substituted product.

Only for the ethynyl-substituted derivatives (using phenylethynylboronic acid pinacol ester) were low yields obtained (Figure 51).

A comparison of platinum and palladium catalysts used in Suzuki couplings clearly shows that platinum complexes are much less active than palladium complexes [72]. A significant advantage of platinum catalysts is that they are inactive in the homocoupling of arylboronic acids. This is an undesirable process that often reduces the chemoselectivity of Suzuki cross-coupling.

## 11. Arene C–H Borylation

Borylation of Arene C(sp^2^)−H provides a rapid and versatile route to synthetically useful (hetero)arylboronic derivatives. A series of catalytic systems has been shown to permit effective undirected and directed borylation of arenes. The complexes of many TMs and rare earth metals have been demonstrated to exhibit catalytic activity in this process. The process has been described in several review articles [73,74,75].

**Figure 52:**
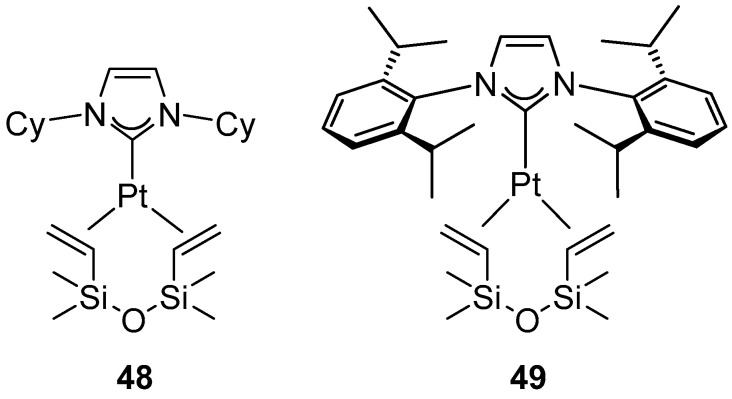
Markó catalysts used in arene C–H borylation [Pt(ICy)(dvtms)] (**48**) and [Pt(IPr)(dvtms)] (**49**).

The first example of the activity of platinum complexes in C–H borylation of arenes and heteroarenes was reported by Tobisu and Chatani [76]. No or low activity was exhibited by simple platinum catalysts, e.g., Karstedt catalyst, as well as phosphine complexes. In contrast, Markó-type platinum complexes with NHC [Pt(NHC)(dvtms)] (Figure 52) allowed the formation of products, with the activity of the complexes depending largely on the stereoelectronic properties of the NHC ligand. In the boronation of monosubstituted benzenes, [Pt(ICy)(dvtms)] (**48**) (Figure 52) was found to be the most active, whereas [Pt(IPr)(dvtms)] (**49**) was completely inactive despite its well-documented high performance in numerous other catalytic transformations. Among the boron reagents tested, B_2_pin_2_ proved to be the most effective, whereas HBpin exhibited only low activity.

**Figure 53:**
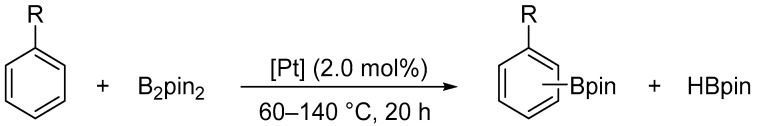
Borylation of monosubstituted benzenes.

**Figure 54:**
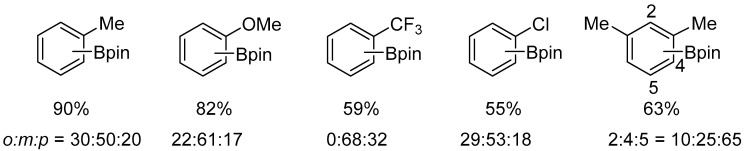
Borylation of mono- or disubstituted benzenes. Reaction conditions: [Pt(IPr)(dvtms)] (**49**) (2 mol%), B_2_pin_2_ (0.3 M), arene used as solvent, 120 °C. The ratio of regioisomers was determined by ^1^H NMR spectroscopy or GC analysis.

The platinum complex [Pt(ICy)(dvtms)] (**48**) catalyzed the borylation of a broad range of arenes bearing electron-withdrawing and electron-donating substituents, including toluene, anisole, chlorobenzene, and trifluoromethylbenzene. This reaction resulted in the formation of a mixture of regioisomers (Figure 53 and Figure 54).

**Figure 55:**
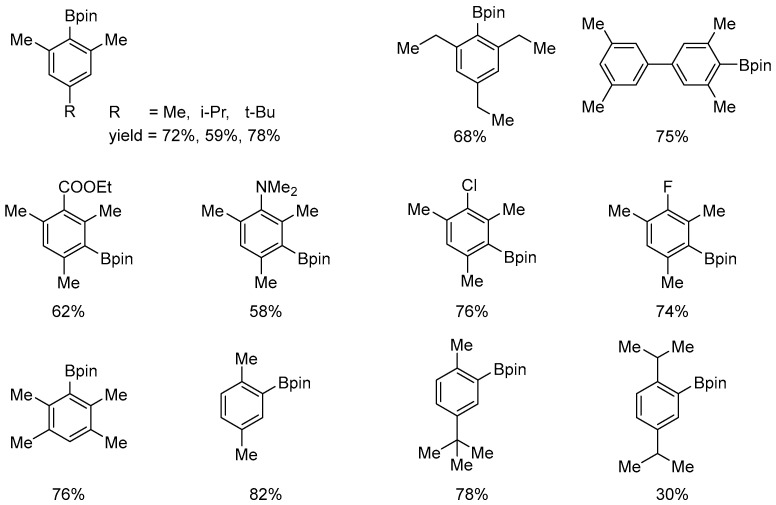
The substrate scope for sterically congested arenes. Conditions: cat. [Pt(ICy)(dvtms)] (**48**), B_2_pin_2_ (0.30 M), arene (solvent), 120 °C.

A notable feature of platinum complexes, particularly [Pt(IPr)(dvtms)] (**49**), is their high tolerance of steric hindrance by arene reagents. This property has enabled the borylation of 1,3,5-trisubstituted arenes to produce the corresponding 2,6-disubstituted phenylboronic ester derivatives (see Figure 55).

**Figure 56:**
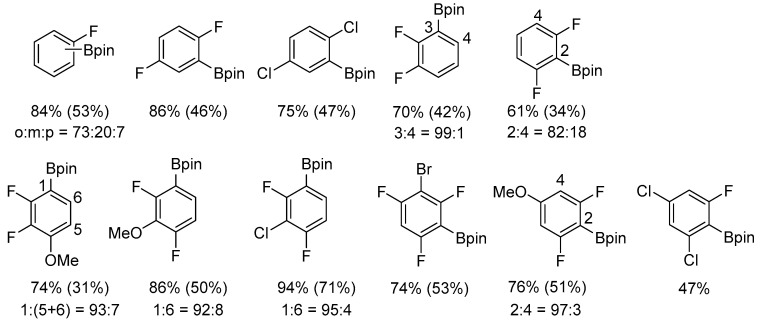
The scope of borylation of fluorobenzenes. The numbers indicate NMR yields (isolated yields in parenthesis) and selectivities. Conditions: [Pt(ICy)(dvtms)], B_2_pin_2_ (2.0 M), arene (5 equiv.), 60 °C or 80 °C. The ratio of regioisomers was determined by ^1^H NMR spectroscopy or GC analysis.

Another key feature of arene C–H borylation catalyzed by platinum NHC complexes is the *ortho*-directing effect of fluorine substituents, enabling the synthesis of *ortho*-fluorophenylboronic ester derivatives. In the presence of [Pt(ICy)(dvtms)] (**48**), high yields and pronounced selectivity towards *ortho*-borylated products were achieved (Figure 56).

**Figure 57:**
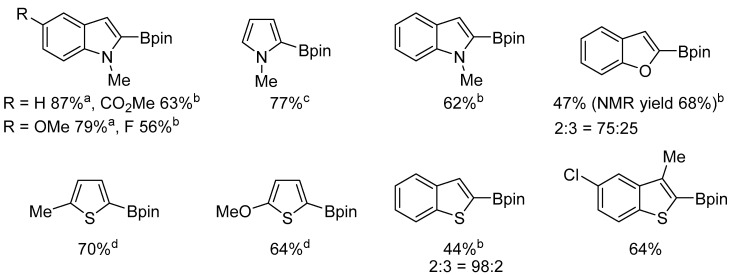
The scope of platinum-catalyzed borylation of heteroarenes. Conditions: [Pt(ICy)(dvtms)] (**48**), heteroarene (0.8 equiv., 0.3 M), heptane, 100 °C. ^(a)^ B_2_pin_2_ (2.0 equiv. relative to heteroarene); ^(b)^ [Pt(ICy)(dvtms)] (**48**) (4.0 mol%); ^(c)^ N-methylpyrrole (5.0 equiv to B_2_pin_2_) was used. ^(d)^ Heteroarene was used as the solvent. The ratio of regioisomers was determined by ^1^H NMR spectroscopy or GC analysis.

The borylation of a series of heteroarenes, including indoles, pyrroles, and thiophenes, afforded the expected derivatives in satisfactory yields (Figure 57).

**Figure 58:**
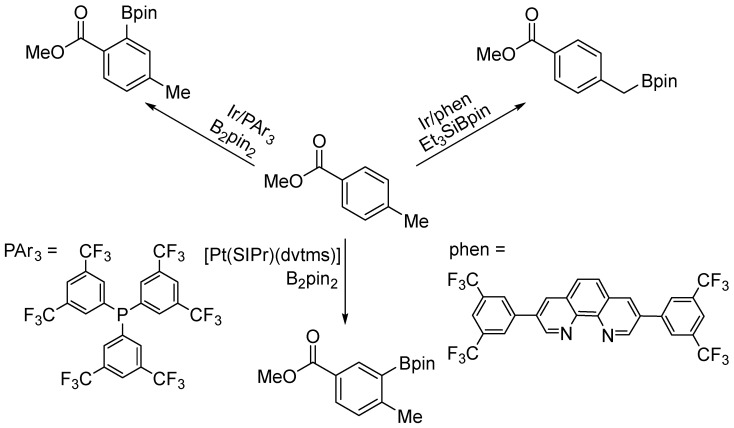
Comparison of site selectivity of hydroboration of methyl 4-methylbenzoate.

A subsequent study by the authors provided numerous additional examples of the hydroboration of sterically congested arenes [77]. A major advantage of this reaction is its ability to furnish sterically demanding 2,6-disubstituted phenylboronic esters, which are difficult to access using existing C–H borylation methods (Figure 58).

**Figure 59:**
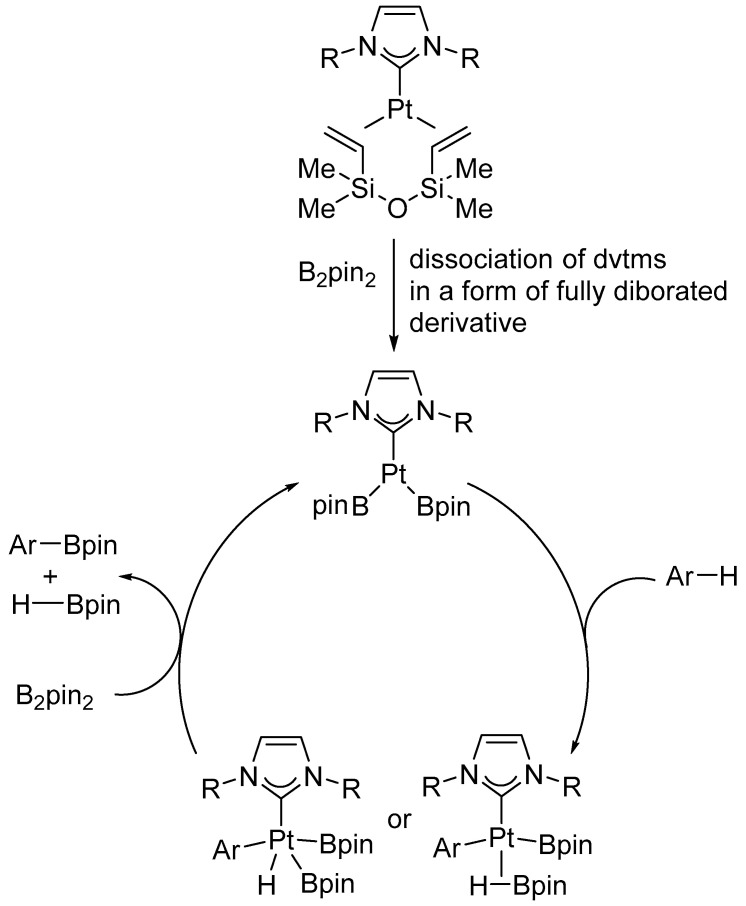
A proposed mechanism for arene C–H borylation catalyzed by NHC platinum complexes [77].

Further studies of the system enabled the authors to propose a reaction mechanism (Figure 59). According to the proposed mechanism, the [Pt(NHC)(dvtms)] pre-catalyst is activated by reaction with B_2_pin_2_. During this process, the dvtms ligand undergoes diboration and dissociates, generating the catalytically active [Pt](Bpin)_2_ species. This complex engages in C–H activation of the arene, with the cleavage of the C–H bond proceeding through either a σ-bond metathesis pathway or an oxidative addition to platinum, consistent with the large KIE observed. The resulting aryl–platinum intermediate subsequently reacts with an additional equivalent of B_2_pin_2_ to furnish the ArBpin product and HBpin, while regenerating the [Pt](Bpin)_2_ catalyst. This mechanistic picture accounts for both the remarkable tolerance toward steric hindrance and the pronounced *ortho*-directing effect observed in fluoroarenes.

Nechaev and Asachenko studied a series of (NHC)Pt(dvtms) complexes as catalysts for the borylation of toluene using B_2_Pin_2_. They observed the unusual ortho-selectivity of C–H activation in toluene, with an isomer ratio of o/m/p reaching ~10:3:1, in the borylation of toluene in the presence of the catalyst (7-Dipp)Pt(dvtms) (**15**, see Figure 15). The reactions were carried out in the presence of 2 mol% of [(NHC)Pt(dvtms)] at 120 °C for 20 h. The stereoelectronic effects of the carbene ligand were shown to significantly impact the catalytic activity and selectivity of the reaction [78]. Reactions carried out under similar conditions using HBpin as a boronating reagent resulted in a significant decrease in regioselectivity.

**Figure 60:**
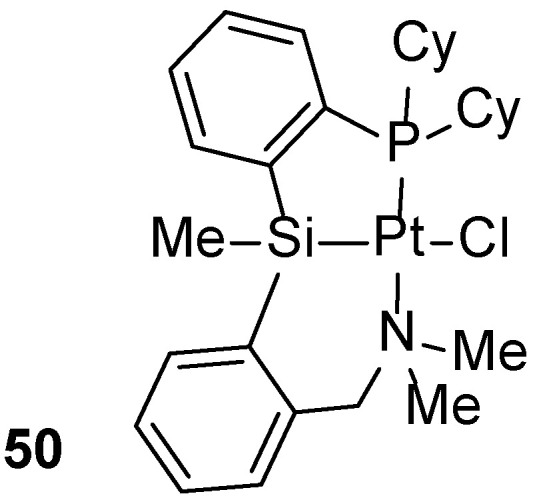
Pincer platinum complex.

Arene C–H borylation can also be accomplished using platinum complexes that do not contain NHC ligands. Iwasawa reported aryl C–H borylation mediated by a non-carbene pincer platinum complex (**50**, Figure 60) [79].

**Figure 61:**
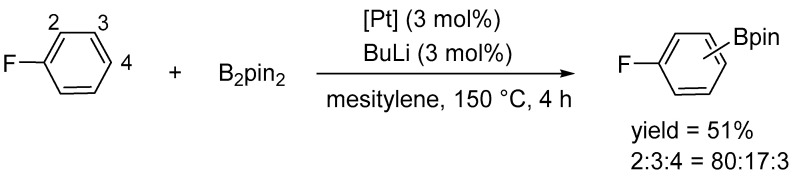
Arene C–H borylation. The *ortho*-directing effect of the fluoro substituent.

The catalyst, similarly to its NHC-containing analogue, exhibits a directing effect of the fluoro substituent toward *ortho*-borylation (Figure 61).

**Figure 62:**
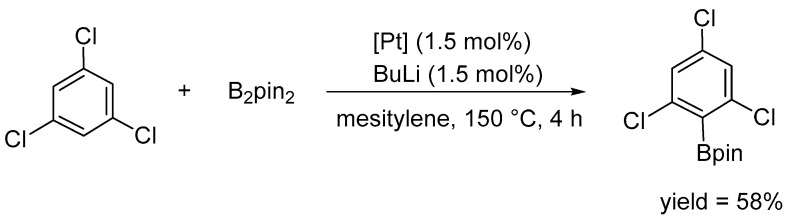
Arene C–H borylation. Insensitivity to steric hindrance.

An insensitivity to steric hindrance is also observed, which enables the borylation of sterically congested arenes (Figure 62).

## 12. Alkyne Hydroarylation

**Figure 63:**
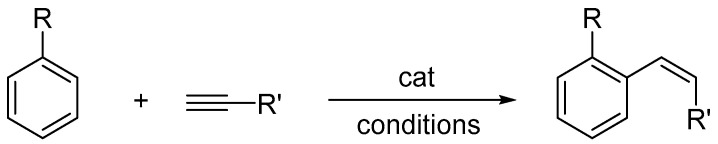
Catalytic intermolecular alkyne hydroarylation.

Dynamic studies on the reaction began with the milestone paper by Fujiwara, who described inter- and intramolecular hydroarylation of alkynes and alkenes with unfunctionalized arenes in the presence of palladium(II) or platinum(II) salts and trifluoroacetic acid (TFA) as the catalyst. The catalytic system enabled efficient regio- and stereoselective intermolecular hydroarylation of both terminal and internal alkynes, often already at room temperature, affording Markovnikov-type addition products in good to very good isolated yields (typically 60–90%) (Figure 63) [80]. To further enhance catalytic activity, PtCl_2_ was also employed in the presence of two equivalents of AgOAc, facilitating the generation of more reactive cationic platinum species and leading to improved conversions.

**Figure 64:**
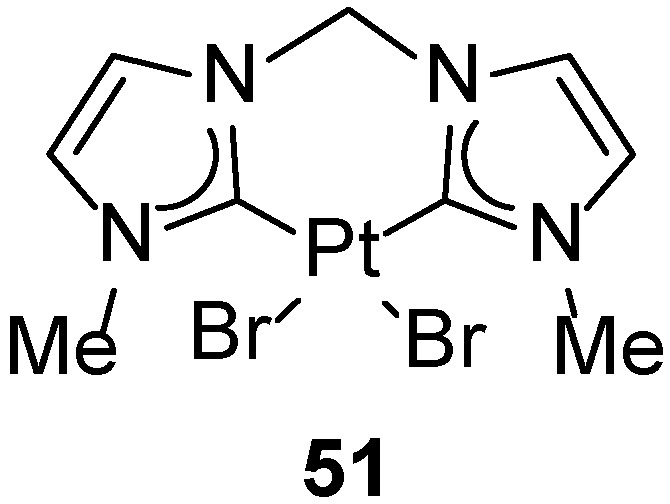
Chelating dicarbene platinum complex (**51**) reported by Biffis as the only example of an NHC-containing platinum catalyst applied in alkyne hydroarylation.

**Figure 65:**
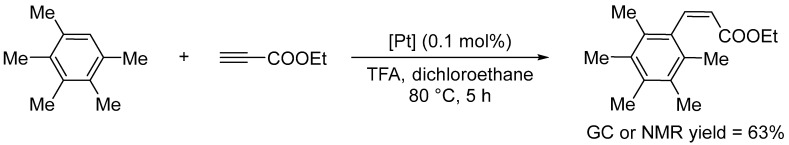
Hydroarylation of ethyl propiolate catalyzed by chelating dicarbene platinum complex.

**Figure 66:**
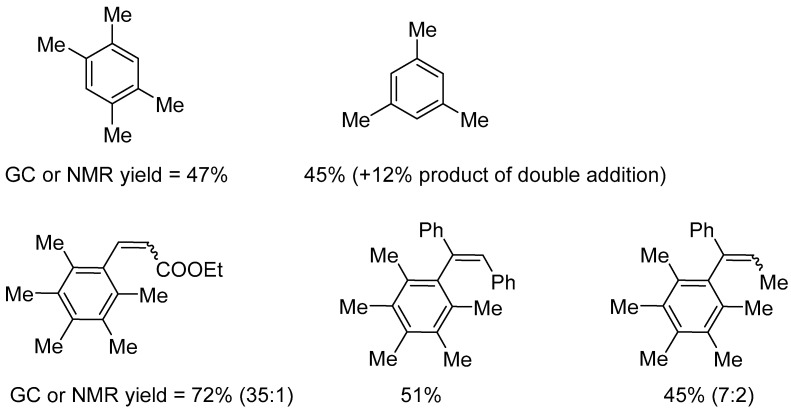
The scope of Fujiwara-type hydroarylation reactions catalyzed by the chelating dicarbene platinum complex (**50**), demonstrating comparable efficiency to the classical PtCl_2_/AgOAc system.

Currently, the reaction can be performed in the presence of numerous transition metal catalysts described in references [81,82,83]. Many platinum(II) complexes, including diisocyanate and cationic ethylene complexes, have been described as catalysts for the hydroarylation of alkynes. Depending on the nature of the alkyne, arene, and reaction conditions used, these reactions can yield the desired products in isolation yields typically in the range of 50–95%, e.g., [84,85,86]. The only example involving NHC platinum complexes in this process was reported by Biffis [87]. The chelating dicarbene platinum complex (**50**, Figure 64) displayed high catalytic activity under the conditions originally developed by Fujiwara, enabling reduced catalyst loadings (0.1 mol%) and elimination of silver additives, while maintaining high chemo- and stereoselectivity and isolated yields typically in the 70–95% range (Figure 65 and Figure 66). Its effectiveness is attributed to the high thermal stability and resistance of NHC ligands under TFA conditions, as well as to their ability to stabilize electrophilic, halide-free Pt(II) species postulated to be the catalytically active forms.

It should be noted, however, that the substrate scope remains largely comparable to that of classical Pt(II) systems and is predominantly restricted to electron-rich arenes, consistent with an electrophilic Friedel–Crafts-type alkenylation mechanism; electron-poor and halogenated arenes are essentially unreactive. In addition, side reactions characteristic of strongly acidic conditions are observed, including Z/E isomerization, ester hydrolysis, and competing hydration or polymerization of terminal alkynes, collectively limiting the synthetic utility of the method.

## 13. Cycloisomerization and Other Cyclizations

Cycloisomerization is a powerful tool for the synthesis of complex molecular architectures from simple reagents. Review articles have been published on the activity of transition metal salts and complexes in cycloisomerisation [44,88]. The activity of platinum complexes has also been reviewed [89,90].

The first report on the use of platinum complexes bearing NHC ligands as catalysts for the reductive cyclization of diynes and enynes is the work of Chung [91].

**Figure 67:**
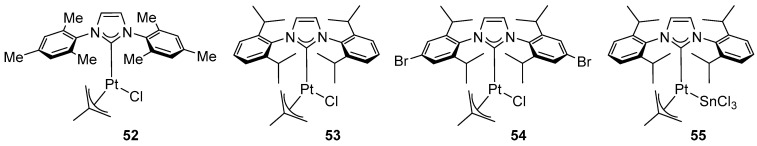
Pt–NHC complexes synthesized and studied by Chung.

**Figure 68:**
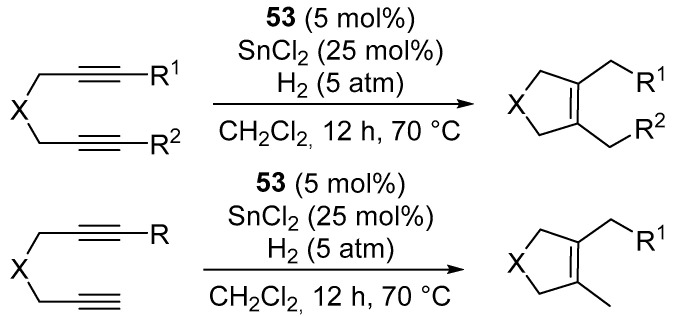
Pt–NHC-catalyzed reductive cyclization of 1,6-diynes.

**Figure 69:**
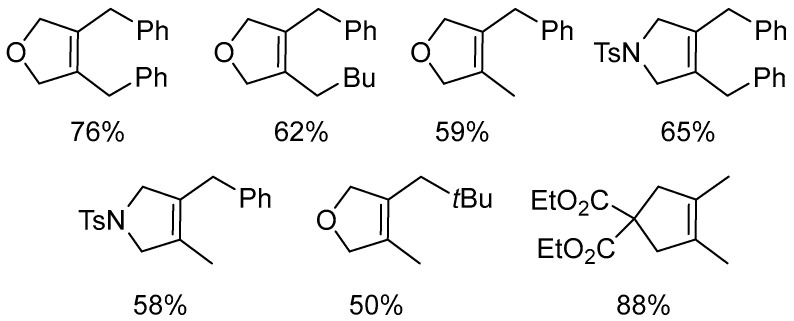
The scope of -Pt–NHC catalyzed reductive cyclization of 1,6-diynes. Isolated yields are given.

The authors described the efficient synthesis of [Pt(η^3^-allyl)(NHC)Cl] complexes (**52**–**55**) (Figure 67), which are stable, easy to isolate, and exhibit exceptional reactivity, differing from the reactivity of previously known platinum systems. The reaction involves the addition of four hydrogen atoms to a diyne, leading to the formation of a 2,5-dihydrofuran (Figure 68). This transformation is exceptionally rare. Previously studied reductive cyclizations of diynes typically afforded only two-hydrogen addition products. The isolation of a stable four-hydrogen-reduced product represents a significant achievement (Figure 69). The work nevertheless presents clear limitations: substrates deviating from the optimal structural architecture often lead to product mixtures or low yields. For example, cycloisomerization of a diyne bearing two terminal ester groups affords the corresponding enyne at only 32% yield, and the formation of a mixture of unidentified by-products. Likewise, extension of the methodology to 1,7-diynes results in a mixture of the primary cyclization product and a further hydrogenated derivative, indicating a loss of selectivity control outside the 1,6-diyne framework.

**Figure 70:**
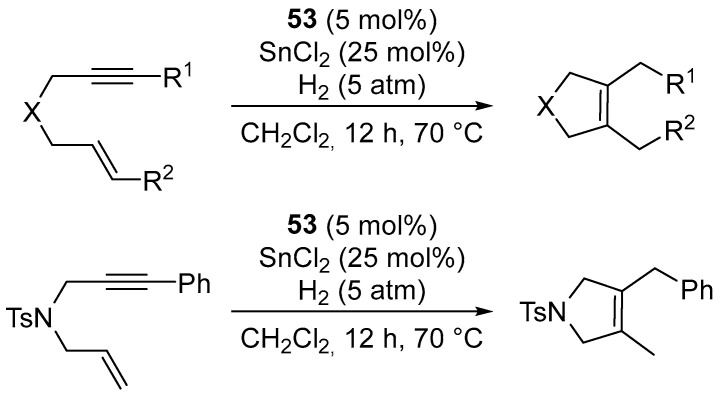
The reductive cyclisation of 1,6-enynes catalyzed by Pt–NHC.

**Figure 71:**
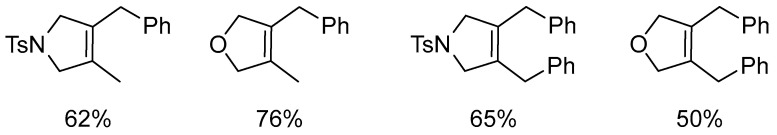
The scope of Pt–NHC-catalyzed reductive cyclization of 1,6-enynes.

Cyclization of 1,6-enynes in the presence of complex **53**, SnCl_2_ and dihydrogen (5 atm) produces 2,5-dihydrofurans and 2,5-dihydropyrroles with medium yields (Figure 70 and Figure 71) [91]. 

Complexes **52**–**55** (Figure 67) exhibit comparable catalytic activity, indicating that the reaction is only weakly sensitive to steric modifications of the NHC ligand. In sharp contrast, the analogous phosphine-based complexes [Pt(allyl)(PR_3_)Cl] (R = Ph, 2-furyl) are completely inactive, even in the presence of SnCl_2_. This observation clearly demonstrates that the NHC ligand does not merely serve a stabilizing role, but actively shapes the energetic landscape of the catalytic cycle. The authors propose that NHC ligands stabilize reactive Pt–H or Pt–(π-alkyne) intermediates, thereby facilitating the hydrometallation cyclization and further reduction sequences while simultaneously suppressing the non-productive deactivation pathways typical of phosphine-based complexes.

Within the broader context of reductive cyclization of 1,6-diynes and 1,6-enynes under hydrogen, a field historically dominated by rhodium catalysis, Pt–NHC systems should be regarded mechanistically and reactivity-wise as complementary rather than directly competitive. Rhodium-based protocols typically furnish so-called “2H” products and operate through distinct mechanistic manifolds that do not involve extensive hydrogen incorporation or monohydride cycles. Palladium has emerged as the metal of choice for enyne cyclization proceeding via hydro- and carbopalladation pathways, yet Pd catalysis rarely enables a formal multi-hydrogen reduction concomitant with cyclization. Against this backdrop, Pt–NHC catalysts occupy a unique niche, offering access to reactivity patterns and hydrogen activation modes that remain largely inaccessible to Rh- or Pd-based systems [92].

**Figure 72:**
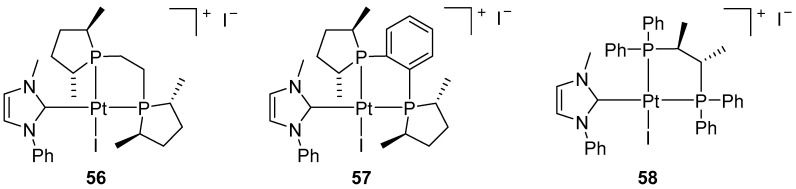
Square-planar axially chiral Pt–NHC complexes proposed by Marinetti.

In 2007, Marinetti proposed a new synthetic approach that enables the preparation of axially chiral platinum(II) complexes with a square-planar structure by combining pure enantiomeric diphosphines (DuPHOS, BPE or Chiraphos) and a non-chiral NHC ligand (Figure 72) [93].

**Figure 73:**
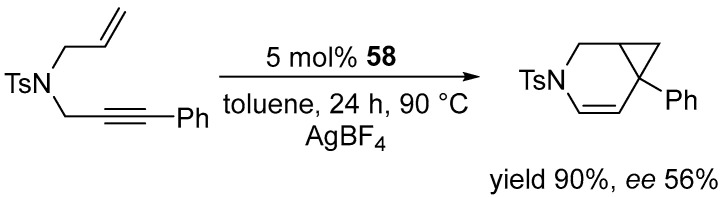
Cycloisomerization of the 1,6-enyne-containing sulfonamide.

The authors demonstrated that the platinum–carbene–diphosphine complexes are competent catalysts for the AgBF_4_-activated cycloisomerization of a 1,6-enyne-containing sulphonamide (Figure 73).

**Figure 74:**
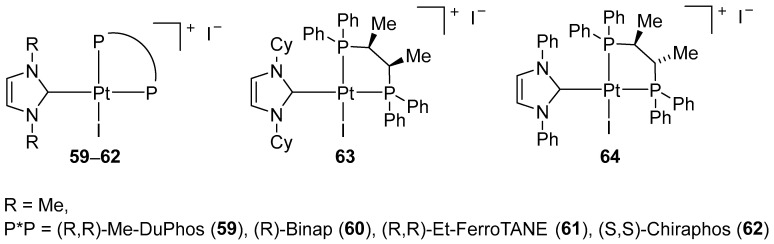
Chiral, non-racemic Pt–NHC complexes.

Although their catalytic activity was slightly lower than that of PtCl_2_ alone, the introduction of chiral combinations of diphosphine/NHC ligands enabled significant asymmetric induction to be achieved. The other complexes consistently provided low levels of enantioselectivity (ee < 15%). In subsequent work, Marinetti expanded the library of square-planar platinum(II) complexes combining chiral, enantiomerically pure diphosphines with NHC ligands. (Figure 74) [94].

**Figure 75:**
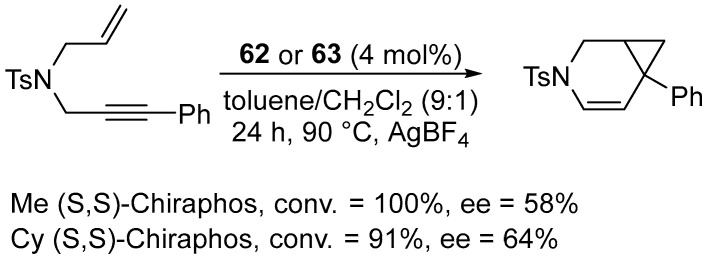
Cycloisomerization promoted by chiral Pt–NHC complexes.

The Pt(II) complexes were tested as pre-catalysts for the cycloisomerization of an allyl-substituted propargylamine derivative (Figure 75).

The results showed yields ranging from 15% to 100%, with enantiomeric excesses of up to 74% being achieved. The highest enantioselectivities were achieved using (S,S)-chiraphos. The combination of an NHC ligand with a chiral diphosphine was proven crucial. The NHC stabilizes the metal center and modulates its electronic properties, while the diphosphine is responsible for chiral induction. Diphosphine complexes without an NHC ligand exhibit poor catalytic activities. However, although the initial square-planar Pt–NHC complexes are configurationally stable, their three-coordinate counterparts readily epimerize under the reaction conditions, indicating that the true catalytic species possesses a dynamic stereochemical character [94].

**Figure 76:**
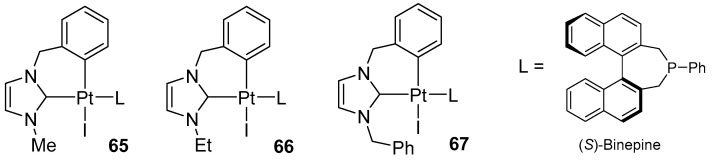
Cyclometallated platinum NHC complexes bearing S-binepine, designed by Marinetti.

The same group designed a family of cyclometallated platinum(II) N-heterocyclic carbene complexes that incorporate an enantiomerically pure monodentate phosphine (Figure 76).

These complexes were used as pre-catalysts in the cycloisomerization of 1,6-enyne derivatives. Tests showed that the skeleton of allylpropargyl tosylamide derivatives underwent highly enantioselective rearrangement when (S)-Ph-binepine was used as a chiral auxiliary ligand.

**Figure 77:**
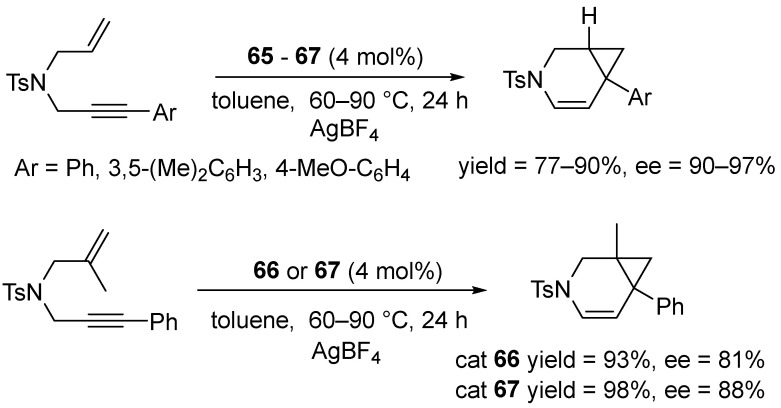
Cycloisomerization of 1,6-enyne derivatives catalyzed by **64**.

Remarkably, an enantiomeric excess of up to 97% ee was achieved (Figure 77) [95].

**Figure 78:**
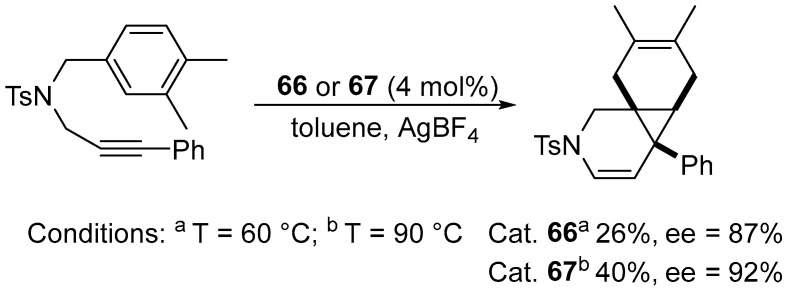
Enantioselective cycloisomerization of enyne promoted by the **66** and **67** complexes.

As an extension of the enantioselective platinum-promoted cycloisomerization strategy, enynes containing a cyclic olefin moiety were used (Figure 78).

**Figure 79:**
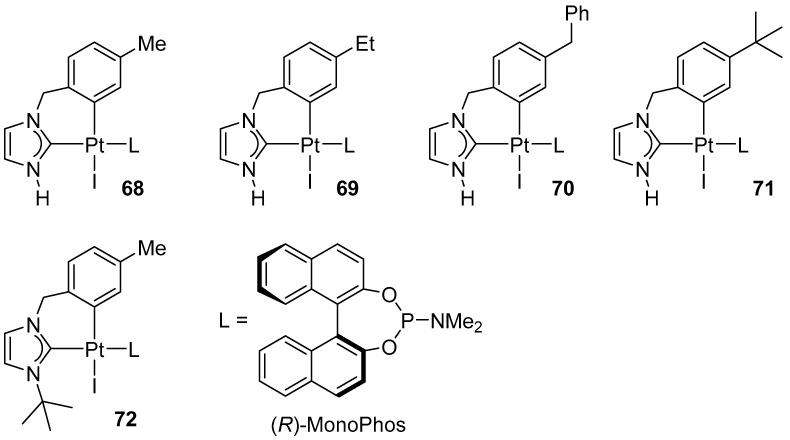
Cyclometallated Pt(II) complexes bearing six-membered NHC ligands and chiral phosphine (MonoPhos).

In 2011, Marinetti expanded the library of cyclometallated Pt(II) complexes with complexes containing six-membered NHC units combined with chiral, monodentate phosphines (MonoPhos) (Figure 79).

**Figure 80:**
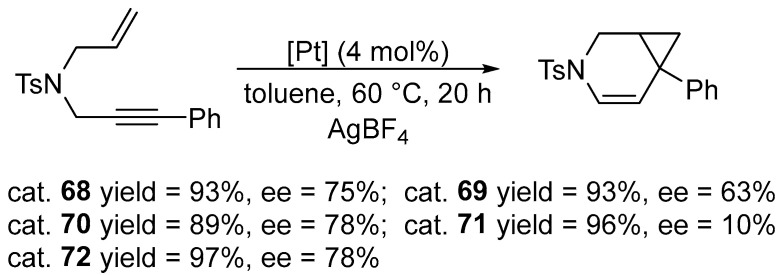
Screening of the (*R*)-MonoPhos-based catalysts 1–5 in the enantioselective cycloisomerization of the model allylpropargylic amine.

These complexes serve as well-defined pre-catalysts for the enantioselective cycloisomerization of nitrogen-containing 1,6-enyne substrates, affording 3-azabicyclo [4.1.0]hept-4-enes at an 89–97% yield and with enantiomeric excesses of up to 78% (Figure 80) [96].

**Figure 81:**
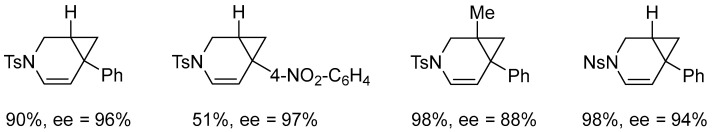
Scope of enantioselective cycloisomerizations of nitrogen-tethered 1,6-enynes catalyzed by **67**.

In parallel, this group synthesized complexes incorporating (S)-Ph-Binepine (**65**–**67**) (Figure 76). Complexes bearing (S)-Ph-Binepine proved to be the most efficient, delivering very high enantioselectivities (88–97% ee) and accommodating a broad substrate scope (Figure 81).

**Figure 82:**
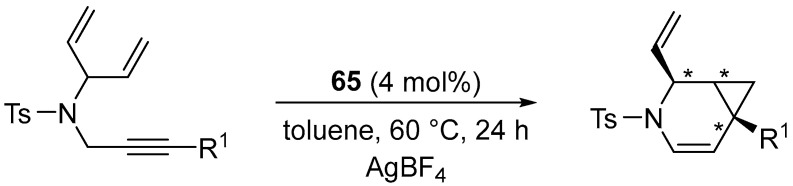
Catalytic cycloisomerization of dienynes in the presence of Pt(II) catalysts.

The described method was extended to prochiral dienes, demonstrating the first examples of enantioselective desymmetrization through cycloisomerization (up to 95% ee with complete diastereomeric control) (Figure 82) [96].

**Figure 83:**
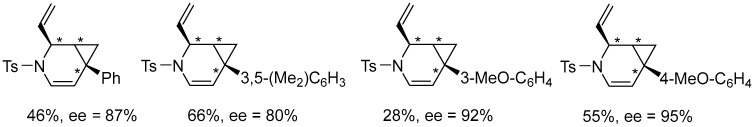
The scope of the cycloisomerization of dienes catalyzed by **65**.

Notably, catalyst **65** afforded very high diastereoselectivity, together with high enantioselectivity, with enantiomeric excesses of between 80% and 95% (Figure 83).

**Figure 84:**
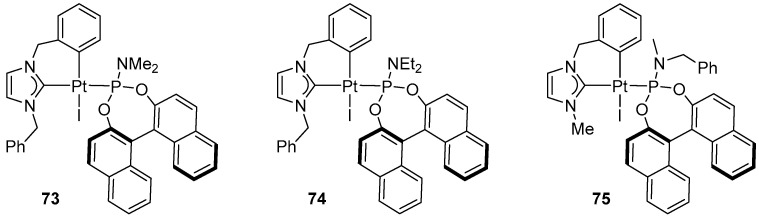
Selected NHC-platinacyclic complexes with phosphoramidite ligand.

Marinetti synthesized a library of Pt–NHC–phosphoramidites (Figure 84) and demonstrated their activity in the enantioselective cycloisomerization of 3-hydroxylated 1,5-enynes [97].

**Figure 85:**
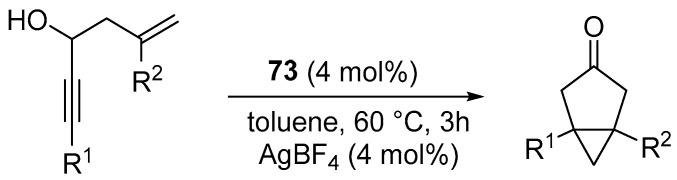
Enantioselective cycloisomerizations of 3-hydroxylated 1,5-enynes catalyzed by **73**.

In the presence of complex **73**, the cycloisomerization of 3-hydroxylated 1,5-enynes (Figure 85) was investigated, demonstrating high catalytic activity and excellent enantioselectivity.

**Figure 86:**
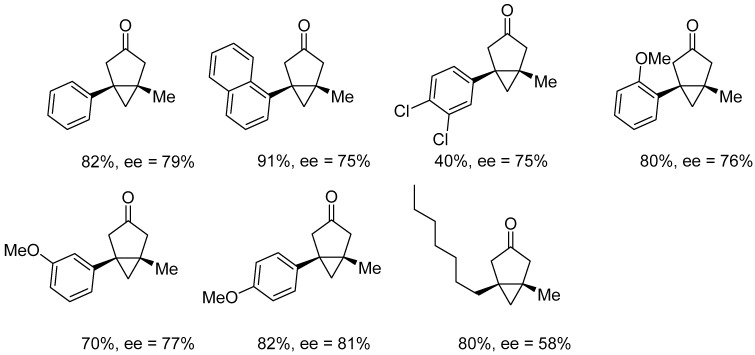
The scope of enantioselective cycloisomerization of hydroxylated 1,5-enynes.

The reactions displayed a broad substrate scope, covering aryl, alkyl, and terminal enynes as well as various types of olefins (Figure 86).

The authors proposed a reaction mechanism in which cyclopropanation constitutes the stereodetermining step. The study suggested that Pt–NHC–phosphoramidites exert full stereochemical control over the reaction [97].

A series of studies published between 2007 and 2013 by the groups of Marinetti, Brissy, Jullien, and Voituriez established platinum(II) complexes bearing N-heterocyclic carbene (NHC) ligands as one of the most thoroughly explored platforms for enantioselective cycloisomerizations of enynes.

The collective studies unambiguously reveal mechanistic constraints that limit the generality of Pt–NHC catalysis. Experimental observations, supported by DFT analyses, indicate that the catalytically competent species is a three-coordinate Pt(II) intermediate formed after halide abstraction. This low-coordinate complex readily undergoes rapid ligand rearrangement, axial inversion, or carbene migration, resulting in partial or complete erosion of the stereochemical information encoded in the pre-catalyst.

A second, equally important limitation is the pronounced dependence on substrate structure. Across all substrate classes examined, optimal enantioselectivities are consistently obtained with aryl-substituted alkynes, which benefit from favorable π-interactions and more defined coordination geometries. In contrast, aliphatic and terminal alkynes typically undergo efficient cyclization but afford only modest or negligible ee values. Similarly, unsubstituted or weakly substituted olefinic components often lead to a dramatic erosion of enantioselectivity, even when chemical yields remain high. In more complex cases, such as dienynes bearing strongly electron-withdrawing aryl substituents, complete loss of reactivity has been observed, underscoring the sensitivity of these systems to electronic effects.

Collectively, these trends indicate that Pt–NHC catalysts are only highly effective within a relatively narrow substrate space where the substrate’s electronic and steric properties align favorably with the chiral environment imposed by the catalyst. Ligand effects further accentuate these limitations. While atropoisomeric BINOL-derived phosphines and phosphoramidites (e.g., Ph-Binepine and related scaffolds) consistently deliver the highest and most reproducible enantioselectivities, other widely used chiral ligands—such as MOP or selected MonoPhos derivatives—often fail to induce asymmetry or exhibit strong substrate sensitivity.

When evaluated against alternative catalytic platforms, Pt–NHC complexes should be regarded as complementary rather than universally superior. Relative to classical platinum catalysts such as PtCl_2_, they offer a decisive advantage in enabling enantioselective variants of cycloisomerization reactions, albeit not always with enhanced rates or lower catalyst loadings. Compared with iridium-, rhodium-, or gold-based systems, Pt–NHC catalysts provide a distinctive combination of structural definition and high enantioselectivity in selected transformations, particularly skeletal rearrangements lacking strong directing groups. However, these benefits are often offset by a narrower substrate scope and greater sensitivity to subtle structural variations.

**Figure 87:**
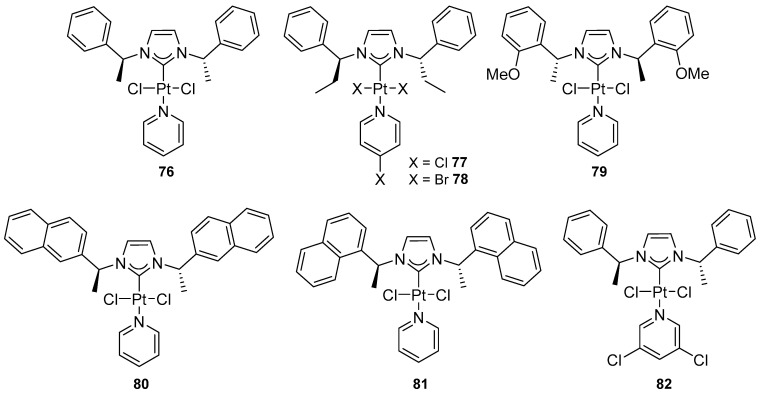
Chiral C2-symmetric NHC platinum(II) complexes reported by Frémont and Blanc.

Frémont and Blanc reported a simple and efficient synthesis of a new series of chiral platinum(II) complexes of the type [Pt(NHC*)(Py)(X)_2_] (**76**–**82**) (Figure 87) [98], which are readily accessible and air- and moisture-stable pre-catalysts.

**Figure 88:**
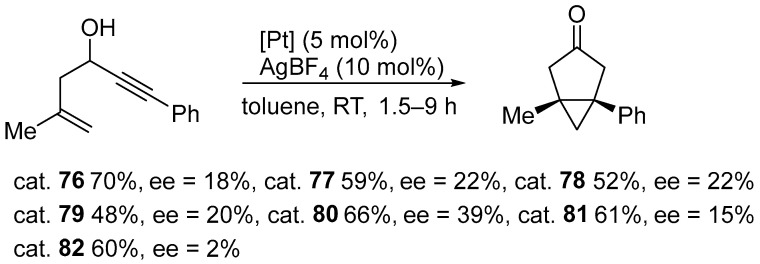
Cycloisomerization of an enynol to the corresponding bicyclo[3.1.0]hexanone.

Complexes were applied as catalysts in the asymmetric cycloisomerization of an enynol to the corresponding bicyclo[3.1.0]hexanone (Figure 88).

Activation with silver salts proved necessary to achieve catalytic activity by generating a vacant coordination site at the platinum center. The complexes exhibited good catalytic performance at room temperature, providing the product at 48–70% yield within 1.5 to 9 h. However, their enantioselectivity remained limited (2–39% ee) (Figure 88). The highest ee values were obtained for the complex containing 2-naphthyl substituents in the NHC ligand. This suggests that increased steric diversity in a chiral environment can enhance asymmetric induction to some extent. By contrast, increasing the acidity of the pyridine ligand accelerated the reaction, resulting in an almost complete loss of stereochemical control [98].

Compared to other catalytic platforms, Pt–NHC complexes benefit from high stability and effective π-activation of enynes. However, their enantioselective performance remains inferior to that of well-established Pd-, Ir-, and, especially, Au-based asymmetric systems, which routinely deliver higher levels of stereocontrol in related transformations [99,100,101].

In conclusion, Pt(II)–NHC complexes emerge as a well-established yet intrinsically specialized platform for asymmetric enyne cycloisomerizations, providing access to high enantioselectivities in transformations that are challenging for conventional platinum or gold catalysts. At the same time, their broader applicability is limited by the configurational lability of low-coordinate Pt(II) intermediates, pronounced substrate dependence, and the need for carefully tailored chiral ligands. Future advances are therefore expected to rely on catalyst designs that more effectively couple the chiral environment to the enantiodetermining step while retaining the characteristic reactivity of platinum(II).

## 14. Perspectives

Based on the well-established understanding of how N-heterocyclic carbene (NHC) ligands influence the catalytic behavior of metal complexes, several beneficial effects can be anticipated: enhanced thermodynamic stability, allowing operation at elevated temperatures; improved reactivity in oxidative addition and reductive elimination steps arising from steric modulation; and increased selectivity of the catalytic processes. The aim of this work was also to examine whether the distinct steric and electronic characteristics of NHC ligands can introduce a qualitatively new dimension to platinum-based catalysis. The results suggest that this is, at least in part, the case. In acetylene dimerization, the presence of an NHC ligand facilitates the activation of the C(sp)–H bond, as well as the reductive elimination of the enyne. Remarkably, this reaction does not occur in the absence of an NHC ligand, underscoring its decisive role in enabling the catalytic cycle. NHC ligands also enhance the capability of platinum complexes to activate strong chemical bonds, such as C–Cl, an effect successfully exploited in Suzuki–Miyaura cross-coupling of aryl and vinyl chlorides with arylboronic acids. Continued progress in platinum catalysis may therefore be anticipated, particularly in transformations governed by successive oxidative addition and reductive elimination events involving the activation of otherwise inert bonds. The unusual regioselectivity of C(sp^2^)–H bond boration of arenes observed in platinum complexes is attractive from a synthetic point of view. The platinacyclic NHC complexes bearing atropoisomeric phosphoramidite ligands developed by Marinetti constitute well-defined, efficient, and modular catalytic systems for the study of platinum-catalyzed enantioselective transformations. In these systems, the NHC ligand serves a stabilizing auxiliary role, though its contribution to the stereochemical outcome of the reaction cannot be ruled out. An important observation, true not only for platinum complexes but also for Pt–NHC complexes, is the strong dependence of the catalytic properties of complexes on the stereoelectronic properties of reagents. The consequence is the need to test a series of NHC complexes with different stereoelectronic properties when searching for the optimal catalyst.

Although there are no such spectacular examples of NHC’s influence on platinum complex catalysis, as seen in Ru–NHC-catalyzed olefin metathesis or Pd–NHC-catalyzed coupling, the progress presented in this article justifies further research into the catalytic potential of platinum complexes containing NHCs and newer-generation ligands.

## Data Availability

Not applicable.
